# Core promoters are predicted by their distinct physicochemical properties in the genome of *Plasmodium falciparum*

**DOI:** 10.1186/gb-2008-9-12-r178

**Published:** 2008-12-18

**Authors:** Kevin Brick, Junichi Watanabe, Elisabetta Pizzi

**Affiliations:** 1 Dipartimento di Malattie Infettive, Parassitarie ed Immunomediate - Istituto Superiore di Sanità, Viale Regina Elena, 299, 00161 Rome, Italy; 2 Department of Parasitology, Institute of Medical Science, The University of Tokyo 4-6-1, Shirokanedai, Minatoku, Tokyo 108-8639, Japan

## Abstract

Little is known about the structure and distinguishing features of core promoters in *Plasmodium falciparum*. In this work, we describe the first method to computationally identify core promoters in this AT-rich genome. This prediction algorithm uses solely DNA physicochemical properties as descriptors. Our results add to a growing body of evidence that a physicochemical code for eukaryotic genomes plays a crucial role in core promoter recognition.

## Abstract 2

A method is presented to computationally identify core promoters in the Plasmodium falciparum genome using only DNA physicochemical properties.

## Background

Eukaryotic promoters are defined as regions containing the elements necessary to control the transcriptional regulation of genes. Typically, a promoter is organized into three regions. The core promoter (CP) spans the region approximately 35 bp upstream of the transcription start site (TSS) and is the binding region for the transcription initiation complex; the proximal promoter, which may contain several transcription factor binding sites, can range for hundreds of base pairs upstream of the TSS; finally, the distal promoter, which may contain additional regulatory elements, such as enhancers and/or silencers, can be located thousands of base pairs from the TSS. The best studied features of the canonical CP are proximal *cis*-acting sequence elements, which have been very well characterized in many organisms. These may include a TATA box, an Initiator element (Inr), a TFIIB recognition element (BRE), and a downstream promoter element (DPE). These sequence elements are, however, by no means ubiquitous, and in fact, it was recently estimated that only a maximum of 20% of mammalian promoters contain a TATA box [1,2].

Much evidence has now emerged showing that epigenetic factors also contribute to transcriptional control of eukaryotic genes [3]. The term epigenetic has been redefined in a modern context as "the structural adaptation of chromosomal regions so as to register, signal or perpetuate altered activity states" [4]. Until recently, it has been difficult to computationally derive these structural adaptations from the DNA sequence; however, the recent work of Segal *et al*. [5] points to the existence of a periodic di-nucleotide 'code' that correlates strongly with nucleosome binding affinity. Interestingly, by using this 'code', it has been shown that nucleosome occupancy at TSS positions in human CPs is very low. Coming at this issue from another angle, it was recently shown that experimentally calculated DNA bendability and a penta-/tetramer based compositional property of DNA exhibit characteristic profiles in the region of TSSs in several higher eukaryotes [6]. These distinctive changes in the conformational profile of DNA around experimentally mapped TSSs reflect local structural traits, which can be considered typical features of CPs. These findings have been corroborated by several other works [7-9], illustrating that profiles of physicochemical properties indeed reveal a TSS specific signal in several eukaryotic genomes.

Despite these recent works into non-motif-based descriptors of CPs, computational methods of promoter identification principally rely on conserved *cis*-acting sequence motifs (in many cases, CpG islands) as descriptors. The extent of this preference is evident from a recent review of promoter prediction programs (PPPs) [10] where all of the eight programs examined use some direct motif/CpG based feature. While in some cases this approach has proven to be very effective [11,12], it is only applicable when the CPs in question are associated with clearly defined sequence elements. In several studies, however, DNA physicochemical properties were incorporated into predictor mechanisms. In the case of McPromoter by Ohler *et al*. [13], the incorporation of a single such parameter into their prediction framework reduced false positive predictions of *Drosophila melanogaster *CPs. More recently, it was shown that by identifying peaks in profiles of DNA structural properties along eukaryotic genomic sequences, CPs could be predicted more accurately than with other PPPs [9]. Furthermore, a PPP was recently developed that used six different physical DNA properties to distinguish between CPs and other DNA sequences, and was shown to outperform 'traditional' PPPs across diverse datasets from eukaryotic genomes [7].

Our interest in prediction methods based on physicochemical properties stems from our studies of promoter regions in *P. falciparum*, the most virulent agent of human malaria, causing millions of deaths globally every year [14]. This parasite is characterized by a complex life cycle that involves two hosts (an invertebrate - mosquito - and a vertebrate - in the case of *P. falciparum*, human) and several morphologically different stages. Such complexity implies dynamic transcriptional control of gene regulation; however, very little is known about the transcriptional mechanisms of this parasite (see reviews in [15,16]). While recent studies have begun to shed some light on these processes through the identification of specific transcription factors and their binding sites [17], the general paucity of information coupled with the exceptionally AT-rich genome [18] mean that computational techniques developed for other genomes are of limited use. In fact, the only PPP that has been specifically applied to the *P. falciparum *genome [9] showed poor performance, prompting the authors to suggest that a bespoke solution was required for this organism.

In the present work, we used DNA physicochemical properties to construct profiles of *P. falciparum *CPs around experimentally determined TSSs in the FULL-Malaria database [19]. We observed characteristic maxima/minima in these profiles at the TSS, confirming previous results with similar parameters in other eukaryotes [6,9]. Furthermore, signals around TSSs allowed us to propose that the actual CP occupies a small region from -35 to +1 nucleotides, as in other eukaryotic genomes. Since these signals are extremely weak and obscured by noise when examined on an individual sequence basis, we have developed a predictor based on an ensemble of support vector machines (SVMs; the Malarial Promoter Predictor (MAPP)) that can identify *P. falciparum *CP regions on the basis of their distinct physicochemical properties.

This is the first time that a computational method has successfully been used to identify TSSs in this genome. We demonstrated that MAPP not only distinguishes a large percentage of TSS positions from non-TSS sequences, but can do so with high spatial accuracy, agreeing with experimental results and representing a useful tool for experimentalists and genome annotators. MAPP predictions on a genomic scale give an insight into CP organization in *P. falciparum*, illustrating that physicochemical properties of the DNA are essential for promoter recognition and suggesting that TSSs occur in broad 'transcriptional start areas' rather than at precise start sites. Furthermore, particular promoter arrangements are revealed (bi-directional promoters, antisense RNA transcription, and so on) that might open novel avenues for the investigation of transcription mechanisms in this organism.

## Results and discussion

### *P. falciparum *core promoter regions have typical physicochemical properties

**Figure 1:**
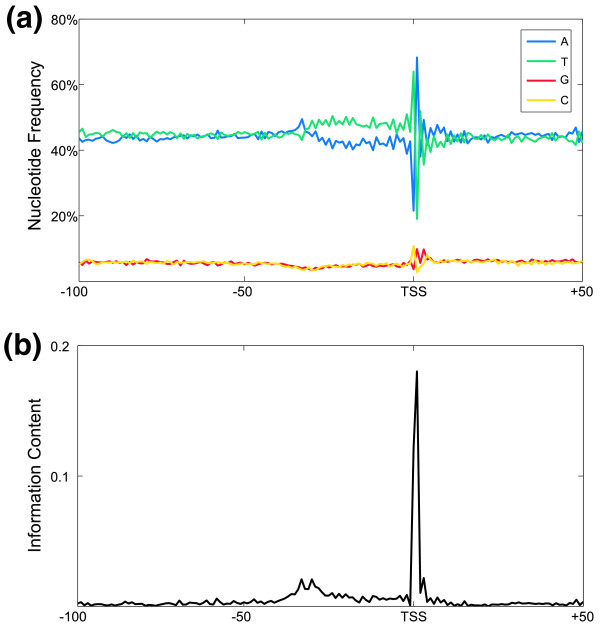
**Sequence conservation at the *P. falciparum *TSS**. **(a) **Nucleotide frequencies in the region from -100 to +50 nucleotides around 3,546 *P. falciparum *TSSs. **(b) **The frequency of each position in the 150 nucleotides around aligned TSS was calculated to generate a position specific scoring matrix. The information content of each position in the matrix was calculated by Σ_*i *_(*p*_i_* log_2_(*p*_*i*_/*b*_*i*_)), where *p*_*i *_= frequency of nucleotide *i *at that position and *b*_*i *_= background frequency of *i*. Background frequencies were calculated from *P. falciparum *intergenic DNA (*b*_*A *_= 0.42, *b*_*T *_= 0.45, *b*_*G *_= 0.07, *b*_*C *_= 0.06).

In order to analyze the composition and conservation of the *P. falciparum *CPs, we extracted sequences spanning 100 nucleotides upstream and 49 nucleotides downstream of each of the 3,546 experimentally mapped TSSs in the FULL-Malaria database [19,20]. This dataset contains at least one TSS for 27% of *P. falciparum *genes. We then aligned these sequences at the TSS and generated a position weight matrix. From this position weight matrix, we calculated nucleotide frequencies and information content at each position around the TSSs (Figure 1). We observed that thymine-adenine is the sequence highly favored at the TSS (Figure 1a), while immediately upstream, for approximately 30 nucleotides, thymine is the preferred nucleotide. Interestingly, the preference for T-A at the TSS reflects the pyrimidine-purine feature (PyPu) present at the TSS in other eukaryotes [21,22], albeit in an AT-rich form (the consensus for the PyPu feature is generally C(G/A), as opposed to the strong TA preference seen here). The PyPu feature at the TSS is generally conserved across different promoter classes [23] and has been shown to be necessary for TFII_D _binding in promoters lacking well defined *cis*-elements [24]. While this feature clearly emerges, the corresponding peak in information content (0.2 bits; Figure 1b) indicates that CPs in *P. falciparum *are characterized by weak sequence conservation. We thus hypothesized that rather than sequence elements, other factors related to the conformation of the DNA molecule may play a role in transcription initiation. This hypothesis is supported by recent evidence in other genomes [6-9].

We used 59 experimentally determined physicochemical properties of DNA (Additional data file 1) in this analysis, along with two different measures of GC content and with the composition based LD parameter of Bultrini *et al*. [6]. Since these properties are based on di-, tri- and tetra-nucleotide sequences, they may reflect similar physical characteristics so that correlations among them must be considered. To do this, we performed a redundancy reduction step (see Materials and methods for details) that resulted in the removal of 28 highly correlated properties. Together with the tetra-nucleotide property (LD), this process yielded a set of 33 non-redundant physicochemical properties that was used in further work.

**Figure 2:**
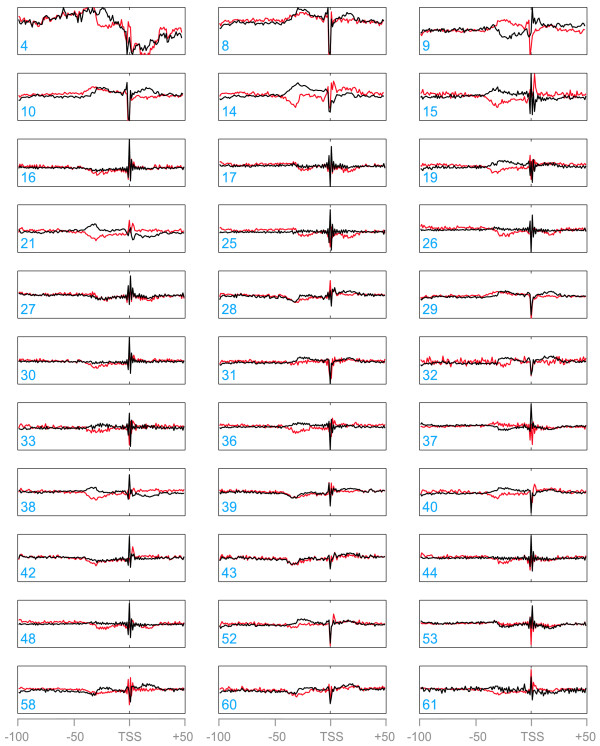
**DNA physicochemical property profiles around *P. falciparum *TSSs**. All 150 nucleotide CP sequences were aligned at TSS positions. For each of 33 non-redundant DNA properties (identified by a progressive number; Additional data file 1), the average profile over the 3,546 sequences was calculated. The average profile is shown for each profile as a black line, and the standard deviation as a red line.

We generated a profile for the 33 selected properties along the 150 nucleotide sequences around each of the 3,546 experimentally mapped TSSs. We used a window size of 2, 3 or 4 nucleotides for di-, tri- or tetra-nucleotide properties, respectively, along with a shift of 1 nucleotide. The normalized average and standard deviation of the profiles are shown in Figure 2 for each of the non-redundant properties. Averaged profiles show characteristic features in a restricted area around the aligned TSSs, and in many cases a corresponding low standard deviation is also observed. Even though, in nearly all cases the strongest 'signal' is seen precisely at the TSS, an additional signal with a low standard deviation is seen approximately 35 nucleotides upstream of TSSs in profiles generated using properties 14, 15, 19, 28, 32, 38, 39, 40, 43 or 60.

The agreement between signals from compositional and physicochemical properties paints a picture of the CP in *P. falciparum*, suggesting that, as is the case for canonical eukaryotic CPs, important features are contained in the short region between -35 nucleotides and +1 nucleotide.

### Support vector machine training with core promoter physicochemical profiles

SVMs comprise a class of supervised machine learning algorithms that can, in principle, separate any two classes of objects. SVMs have been applied extensively to bioinformatic problems from analyses of microarray data to protein fold recognition (for comprehensive reviews, see [25,26]). Recently, SVMs were successfully applied to detect sequence based biological signals in the human genome, including characteristic motifs at the TSSs [27].

We decided to construct a predictor combining SVMs trained to recognize CPs in the *P. falciparum *genome on the basis of signals observed in the 33 physicochemical profiles. First of all, we carefully selected sequences (positive and negative data) for training and testing the SVMs. We used sequences from -100 to +49 nucleotides around each experimentally determined TSS as positive data [19,20]. Negative data were generated by selecting 150 nucleotide sequences from both intergenic (IG) and exonic (EX) genomic DNA (from version 2.1 of the genome). Since IG sequences may contain distal or undocumented TSSs, we used the length distribution of 5' untranslated regions derived from *P. falciparum *full-length cDNAs (flcDNAs) to establish criteria for IG selection. Having observed that only 3.2% of the transcripts begin at a distance greater than 2,000 nucleotides from the closest gene, we decided to select IG sequences that were at least 2,000 nucleotides away from any annotated gene. Excessive false positive predictions is one of the greatest problems for CP predictors, and thus, we used a CP:IG:EX ratio of 1:2:2 during the training (Table 1). The remaining sequences were divided into two independent test sets, the smaller test set (Test 1) was used to find the optimal combination of SVMs for the final predictor (see below), while the larger test set (Test 2) was used to assess the final predictor.

**Table 1:** Number of sequences used for SVM training and testing

	CP	IG	EX
Training	1,100	2,200	2,200
Test 1	610	302	302
Test 2	1,834	910	910

CP, number of core promoter sequences; IG, number of intergenic sequences; EX, number of exonic sequences.

Sequences were converted into physicochemical profiles and a SVM was trained for each of these properties. Some positions in physicochemical profiles (features) may not contribute to prediction ability and, hence, may reduce performance and increase the computational burden. For these reasons we used a wrapper-type feature selection algorithm (for details see Materials and methods) to establish positions in physicochemical profiles that best discriminate CPs from negative sequences. The relevance of each position around the TSSs was evaluated, then different combinations of the most relevant ones were used to train a SVM with fivefold cross-validation. For each set of selected positions, the SVM performance was evaluated and the combination that gave optimal fivefold cross-validation accuracy during the training process was chosen (see Materials and methods; Additional data file 2). Even though this selection strategy considers positions independently, the process only results in the removal of features that have a net detrimental effect on SVM performance.

**Figure 3:**
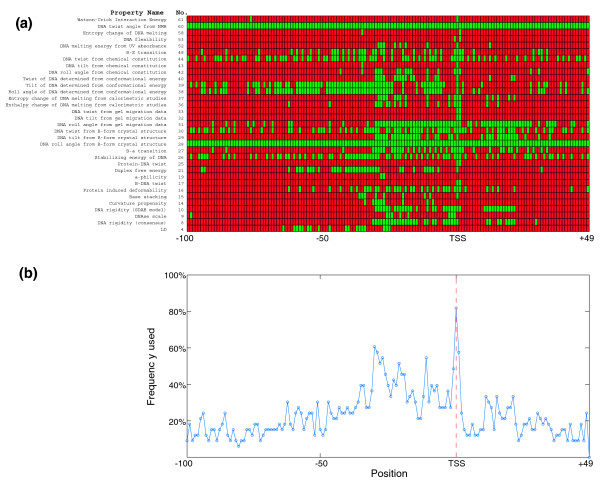
**Frequencies of features used by SVMs for training**. **(a) **The features used for training each SVM. Green boxes indicate features used to train an SVM with that physicochemical property. Red boxes indicate features that were not used. **(b) **The relative frequency with which each feature is used in SVM training highlights the most important positions for accurate SVM training.

Besides reducing the computational cost and improving SVM performance, the results of this feature selection are interesting *per se *as they show the localized importance of each physicochemical feature around the TSS. In Figure 3a, the optimal set of features for training each SVM are shown (selected features are green, unselected are red). From these, a complex picture of the local physicochemical properties at the *P. falciparum *CP emerges. Some notable patterns of biological significance could be identified. For example, we observed that in the region between -31 nucleotides and the TSS, DNA rigidity is an important consideration (properties 8 and 10; 49/62 features are used). The entropy (properties 37 and 52) and enthalpy (property 36) upon 'melting' of this region are also distinctive, particularly in the 5' region, close to the -31 nucleotides position. These results in combination with profiles in Figure 2 suggest that while rigid, this region may be easily zipped open when required for transcription. The results for the protein-induced deformability (property 16) are also particularly interesting. Selected positions are from -64 to +30 nucleotides, suggesting that this entire region may be particularly amenable to binding of general transcription factors (such as TFII_D_) that deform the DNA when they bind.

Despite the complexity of these results, when we analyzed the frequency with which each feature is used in overall SVM training (Figure 3b) a clearer pattern emerged. The most frequently used features are found precisely at the TSS (0 to +1; used to train 81% and 60% of SVMs, respectively) and in the region from -35 to -20 nucleotides upstream of the TSS.

### Consolidation of SVMs into the MAPP

In order to assess which of the SVMs gave the best performance, we utilized the first test dataset (Test 1). In addition to specificity and sensitivity, we also calculated the harmonic mean (F) as this measure equally weights type I (false positives) and type II errors (false negatives) (see Materials and methods). The performance for each of the 33 SVMs is reported in Table 2. The most robust single classifier (F = 0.52) is that trained with property 60, the twist of DNA, as determined by NMR [28]. This classifier has the highest sensitivity of all SVMs (0.37), yet the specificity is somewhat low (0.97). Other SVMs, such as that trained with property 14 - AT and GC type curvature propensity [29] - correctly predict fewer promoters (sensitivity = 0.09), but have a specificity of 1.00, meaning that IG and EX sequences are never predicted as CP. Nine trained SVMs were unable to distinguish CP from negative sequences and, thus, have no predictive value (sensitivity = 0, specificity = 1). These nine SVMs were discarded and not used in subsequent steps. MAPP combines the outputs of the remaining 24 trained SVMs to give a prediction. We trained a final SVM to combine these outputs in order to derive a single MAPP score (between 0 and 1) for each sequence.

**Table 2:** Cross-validated SVM performances

Property	Sensitivity	Specificity	F-score
4	0.10	0.99	0.17
8	0.21	0.99	0.35
9	0.18	0.99	0.30
10	0.28	0.98	0.43
14	0.09	1.00	0.17
15	0.25	0.97	0.39
16	0.28	0.95	0.42
17	0.00	1.00	0.00
19	0.00	1.00	0.00
21	0.20	0.97	0.32
25	0.00	1.00	0.00
26	0.34	0.96	0.49
27	0.18	0.98	0.30
28	0.32	0.96	0.48
29	0.27	0.98	0.41
30	0.30	0.96	0.45
31	0.30	0.96	0.45
32	0.00	1.00	0.00
33	0.00	1.00	0.00
36	0.24	0.97	0.37
37	0.24	0.96	0.38
38	0.29	0.96	0.43
39	0.25	0.95	0.38
40	0.12	0.99	0.21
42	0.12	0.98	0.21
43	0.00	1.00	0.00
44	0.19	0.97	0.32
48	0.33	0.95	0.48
52	0.09	0.99	0.16
53	0.00	1.00	0.00
58	0.00	1.00	0.00
60	0.37	0.97	0.52
61	0.00	1.00	0.00

The performance of each of the SVMs after cross-validated training using each individual physicochemical property of DNA.

For each combination of the top *n *SVMs as ranked by F-score ({*n*|*n *∈ *Z*, 1 ≤ *n *≤ 24}; Table 2) we calculated the area under a receiver operating characteristic (ROC) curve (AUC). This is a useful single figure representation of overall performance for which random choice will yield an AUC of 0.5, while a perfect predictor will yield an AUC of 1.0. By combining individual predictions, the AUC is increased from 0.835 to 0.883, with the maximum AUC achieved using 17 SVMs. The AUC saturates after n = 17, yielding similar AUCs for all combinations up to the maximum of n = 24. The cumulative effect confirms that the physicochemical properties selected to train SVMs provide independent and complementary information on the CP in *P. falciparum*. To generate the final MAPP score (M_sc_), we chose n = 21, a point in the middle of the optimal range.

### MAPP assessment

**Figure 4:**
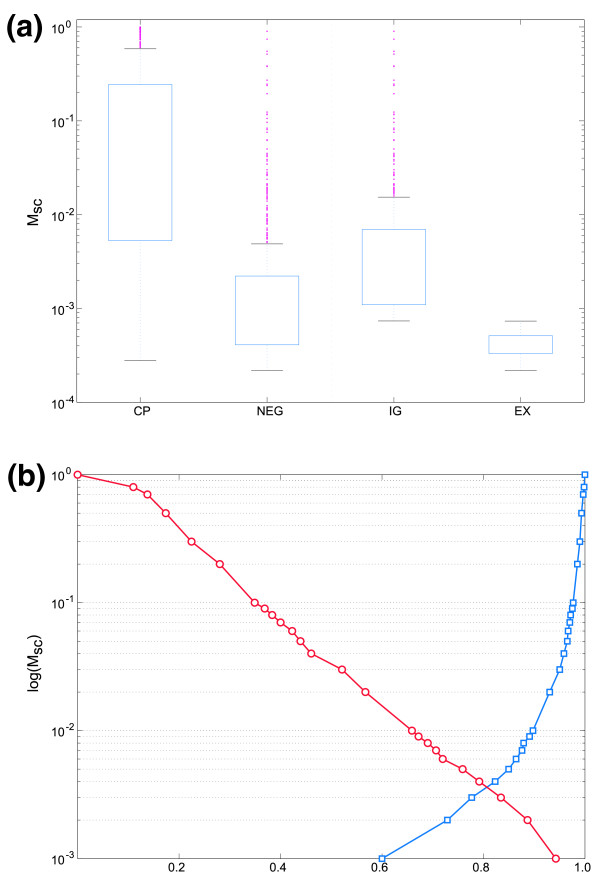
**MAPP score distributions**. **(a) **The distribution of MAPP scores (M_sc_) for core promoter (CP) and negative (NEG) sequences are given for the test dataset Test1. Upper and lower limits of the box represent the upper and lower quartiles of the distribution, respectively. Whiskers extending from the boxes represent the extent of the rest of the data distribution, while outliers are represented by magenta points. On the right-hand side of the dotted line is the breakdown of the NEG distribution into separate distributions for intergenic (IG) and exonic (EX) sequences. **(b) **The specificity (blue squares) and sensitivity (red circles) at different M_sc _thresholds.

The performance of the final predictor, MAPP, was assessed on the second test set (Test 2). First of all, we studied the distributions of M_sc _for CP and negative sequences (IG and EX; Figure 4a). The distributions of CP and negative sequences only partially overlap, with most of this overlap due to IG sequences. For M_sc _higher than 0.05, few false positives are expected and predictions with M_sc _>0.94 have 100% accuracy. It is more prudent to state the error rate at this threshold as <1 false positive per 910 nucleotides IG DNA, and <1 false positive per 910 nucleotides IG DNA.

A more detailed analysis reveals that a clear and highly significant (*p *< 10^-100^, Wilcoxon rank sum test) difference is seen between the mean of the CP M_sc _(${\bar{M}}_{sc}$ = 0.19 ± 0.30) and the mean of the negative sequence M_sc _(${\bar{M}}_{sc}$ = 0.02 ± 0.09). Interestingly, the three input groups (CP, IG and EX) exhibit statistically different score distributions (*p *< 10^-100^, 3× Wilcoxon rank sum test), despite not having been trained as such. This further separation of the exonic profiles is very likely due to the diverse nucleotide composition of coding and non-coding DNA in *P. falciparum *[18].

Quantitatively, these results are best expressed as specificity and sensitivity. We calculated these values for MAPP predictions at 30 M_sc _thresholds (Figure 4b). At each threshold (*t*), a sequence with M_sc _≥ *t *is considered a TSS prediction. For example, if we consider the most permissive criterion of M_sc _≥ 10^-3 ^(any sequence with a positive M_sc _is considered a TSS), we achieve a sensitivity of 0.94 (red circles) and a specificity of 0.60 (blue squares). By increasing the M_sc _threshold, the specificity increases and exceeds 0.99 at M_sc _≥ 0.6. Notwithstanding that the CP:EX:IG ratio used in these assessments does not reflect the true ratio in the genome (where CP sequences would be far less frequent), the high specificity does indicate that MAPP may be well suited for genomic scale applications.

### Positional effect on MAPP score

In order to assess the positional precision of MAPP, we generated a prediction for every nucleotide position in the region from -400 to +200 nucleotides around each TSS in the Test 2 dataset. At each position in the 601 nucleotide window, we calculated the average M_sc_. We then counted the number of nucleotides adjacent to the TSS for which the M_sc _remained more than one standard deviation above the mean (Additional data file 3). We found this region spans 101 nucleotides almost symmetrically around the TSS. This can be considered the positional accuracy of MAPP prediction. These results, as well as being important to evaluate genome scale predictions of MAPP, are also interesting from a biological point of view. The broad distribution of high M_sc _in the region immediately around TSSs may be due, in part, to the presence of multiple start sites, suggesting the presence of 'transcriptional start areas' from which several transcripts arise. This is in line with the available experimental data for *P. falciparum*; in the three cases of finely characterized promoters [30-32] and for almost half of the genes with mapped TSSs [19], multiple start sites are observed. Furthermore, recent evidence from high throughput studies in mammalian genomes suggests that an 'area' with several TSSs dispersed over tens of nucleotides, rather than a single specific start nucleotide, is the predominant type of promoter architecture [23].

**Figure 5:**
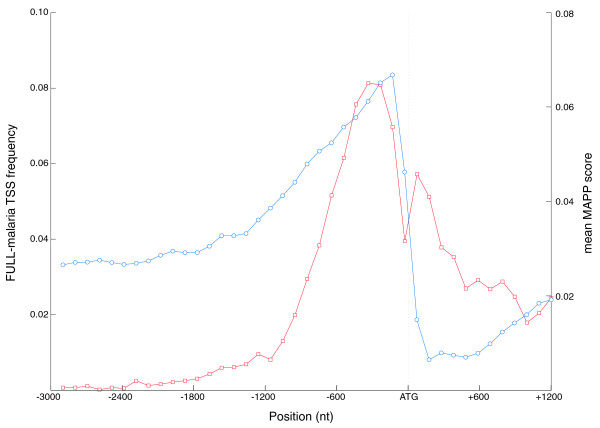
**MAPP score distributions and comparison with experimental TSS distributions**. A MAPP profile was generated for the region from 3,500 nucleotides upstream to 1,200 nucleotides downstream of every gene start codon in the *P. falciparum *genome (v2.1.4). These MAPP profiles were aligned at the 0 position (ATG codon) and the MAPP score averaged at each position. We smoothed the average MAPP score using a sliding window of 200 nucleotides and a shift of 100 nucleotides (blue circles). The TSSs distribution was generated from the frequency of FULL-Malaria TSSs at each distance from the closest ATG codon (red squares). Multiple TSSs that mapped to the same nucleotide were considered as a single mapping.

To assess the positional preferences of predictions relative to gene start codons, we generated predictions for 3,000 nucleotides upstream and 1,200 nucleotides downstream of all *P. falciparum *gene start sites. At each position we averaged the MAPP scores (blue circles in Figure 5). The MAPP score peaks in the 1,000 nucleotide region upstream of start codons. This illustrates a striking preference for strong predictions upstream of ATG start codons. Furthermore, the MAPP distribution from -3,000 nucleotides to ATG is highly correlated with the TSS distribution derived from experimental flcDNA mappings (red squares in Figure 5; Pearson correlation coefficient = 0.96). Immediately 3' to the gene start site, there is a dramatic dip in the MAPP score, confirming that MAPP makes very few TSS predictions in exonic regions.

When predictions are performed on large genomic sequences, MAPP cannot assign predictions to one strand or another. In fact, we observe very similar predictions on both DNA strands but shifted by approximately 40-50 nucleotides from each other (the correlation coefficient between the plus and minus strand profiles for chromosome 14 rises from 0.33 to 0.56 if we shift one of the profiles by 50 nucleotides). As previously shown, those positions in the SVM input vectors that are most discriminative for classifying training sequences are between -35 nucleotides and +1 nucleotide. When this region of an input vector overlaps with a strong promoter signal (that is, -35 nucleotides to +1 nucleotide around a true TSS), a high M_sc _is output at the TSS (position 0 nucleotides; for a detailed schema, see Additional data file 4). However, if the overlap is in the reverse orientation (that is, from +1 to -35 on the opposite strand), a strong, similar M_sc _will result for a nucleotide at the other extreme of this window (position -34 nucleotides). Other, weaker signals (from -50 to +25 nucleotides) account for the variability of the shift size observed between the two profiles. In subsequent analyses, unless otherwise stated, we consider only the MAPP predictions on the same strand as the gene of interest.

### Evaluation with EGASP criteria

The Encode Genome Annotation Assessment Project (EGASP) established a set of standard criteria by which the performance of a PPP can be assessed (see Materials and methods for details) [33]. This assessment was important to give a true reflection of MAPP performance on a genomic scale, where the CP:EX:IG ratio is very different to that used in the SVM training/test processes.

For each gene with an upstream TSS in the Test 2 dataset, we constructed a MAPP profile from the position of the most upstream TSS to the downstream gene stop codon. MAPP predictions were then clustered at different M_sc _thresholds (*t*; for details, see Materials and methods). This simplified each profile into a series of single point predictions (each cluster center is a prediction). In previous studies on other genomes, a maximum allowed distance of ± 500 or ± 1,000 nucleotides between true and predicted TSSs has been commonly used [33]. Given the relative compactness of the *P. falciparum *genome, we decided to consider only maximum distances (*w*) of ± 50 nucleotides and of ± 100 nucleotides. Each analysis was thus extended upstream of the 5' TSS by *w *nucleotides to allow for predictions that fall in this region. In addition to the positive predictive value (PPV) and sensitivity, we also calculated the harmonic mean (F). F equally weights the PPV and sensitivity, ranging from 1 (best performance) to 0 (worst performance), and hence is a useful measure to assess overall predictor performance.

As expected, the MAPP performance was better at each *t *cutoff when we used the ± 100 nucleotide window size (second column in Table 3,). Irrespective of which window size was used, a reduction in the clustering threshold reduced the PPV and increased the sensitivity. In general, it also reduced the F-score, illustrating that the PPV cost outweighed the sensitivity benefit at lower thresholds. We determined that the optimum MAPP clustering threshold as judged by F-score was M_sc _= 1.0 when using a ± 50 nucleotide error window (F = 0.40, PPV = 0.72, sensitivity = 0.28) and Msc ≥ 0.9 when using a ± 100 nucleotide window (F = 0.51, PPV = 0.54, sensitivity = 0.49). In addition, if clusters are derived from only MAPP predictions with a M_sc _= 1, the PPV at each window size is >0.7 (PPV_50 _= 0.72; PPV_100 _= 0.80). As a result of these high PPVs, we can have a very high confidence in such MAPP predictions on genomic scale as they guarantee a very low number of false positive predictions. It should also be noted that we probably underestimated MAPP performance in this evaluation. Specifically, our evaluation over-counts false positive predictions as the FULL-Malaria database does not provide a complete representation of TSSs for a gene. This is evidenced by the fact that 73% of *P. falciparum *genes do not have a 5' mapped TSS. Furthermore, several studies have identified TSSs that are absent from this dataset [30,31].

**Table 3:** MAPP performance by EGASP criteria

*t*	Sn_50_	PPV_50_	F_50_	Sn_100_	PPV_100_	F_100_
1.0	0.28	0.72	0.40	0.36	0.80	0.49
≥ 0.9	0.34	0.41	0.37	0.49	0.54	0.51
≥ 0.8	0.35	0.35	0.35	0.52	0.46	0.49
≥ 0.7	0.37	0.32	0.34	0.55	0.43	0.48
≥ 0.6	0.37	0.29	0.32	0.56	0.41	0.47
≥ 0.5	0.37	0.27	0.31	0.57	0.38	0.46
≥ 0.4	0.36	0.24	0.29	0.57	0.36	0.44
≥ 0.3	0.35	0.22	0.27	0.59	0.34	0.43

The performance of the MAPP was assessed using the criteria designed for the EGASP promoter prediction workshop. Each analysis was run with a TP window acceptance size (*w*) of ± 50 nucleotides or ± 100 nucleotides. *t*, MAPP score clustering threshold; PPV_*w*_, positive predictive value; Sn_*w*_, sensitivity; F_*w*_, harmonic mean.

From the M_sc _distributions in Figure 4a., we would have expected very few TSSs to have a MAPP score ≥ 0.6 (specificity = 0.17). Apparently, this is in contrast to the MAPP specificity established with EGASP criteria (specificity = 0.37). This can be explained by the imprecision of flcDNA mappings or by the presence of more TSSs than we know of. flcDNAs are generated by a system that also has an implicit error. It has been shown that 7.2% of TSSs derived from flcDNA in the Database of Transcriptional Start Sites (DBTSS) were more than 100 nucleotides distant from equivalent mappings in the Eukaryotic Promoter Database (EPD) [34].

We also compared the performance of MAPP with the only other PPP that can be justifiably applied to the *P. falciparum *genome (EP3) [9]. EP3 is, however, known to perform relatively badly in this organism compared to others. We confirmed that EP3 was not effective at identifying promoters at either window size (± 50 and ± 100 nucleotides) as in both cases it yielded PPV, sensitivity and F-scores below 0.02.

### Validation with independent experimental data

We performed some independent analysis of the quality of our predictions with data not derived from the FULL-Malaria database. In this way, we could also assess the empirical usefulness of our predictions on a gene-by-gene basis. We identified independently mapped TSSs in the literature and selected the upstream regions of three representative cases for this validation (the others are illustrated in Additional data file 5). For each nucleotide in the selected regions, a MAPP score was calculated and predictions are shown as a plot along the genomic sequences (MAPP profile).

#### *PF11_0009 (rifin)*

**Figure 6:**
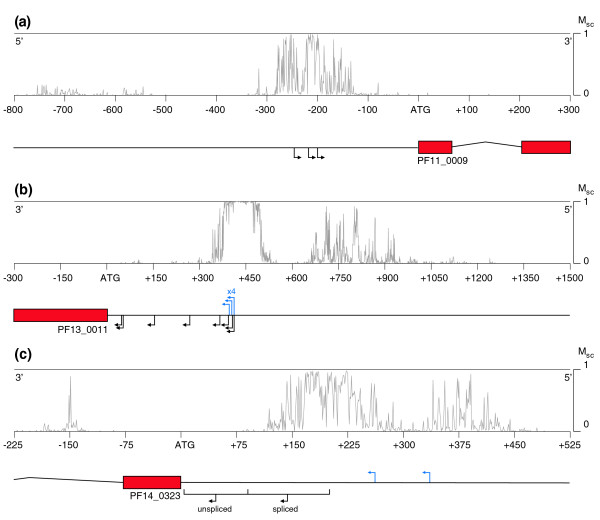
**TSS predictions are consistent with independent experimental data**. MAPP predictions for the same strand as the studied gene are plotted above the genome annotation. **(a) ***PF11_0009*; **(b) ***PF13_0011*; **(c) ***PF14_0323*. The MAPP profile ranges from 0 to 1 (maximum). Red rectangles represent genes and arched lines represent introns. The genome is represented by the black line upon which each gene is centered. Blue arrows above the genome line represent TSSs from the FULL-Malaria database, while black arrows below the genome line are those that have been identified in other studies. Numbers above these arrows are the number of multiple TSS that could not easily be distinguished at the scale with individual arrows. In all cases, only one DNA strand is shown and directionality can be inferred from the direction of TSS arrows. The scale is given between the genome and the MAPP profile and is zeroed at the translation start site of the gene. In (c), the combined regions represented by the parentheses contain 47 individual TSSs. Those TSSs between the start codon and -80 nucleotides predominantly give rise to unspliced transcripts, while those in the region further upstream (to -172 nucleotides) give rise to correctly spliced mRNA.

The upstream region of the *rifin *gene *PF11_0009 *was recently characterized experimentally [31]. In this work, TSSs were mapped using 5' RLM-RACE and it was shown that transcription initiates from three positions in a 47 nucleotide window (-198, -216 and -245 nucleotides; black arrows in Figure 6a). The MAPP profile peaks in the regions around all three mapped TSSs, with maximum M_sc _(M_sc _= 1) at the locations of TSSs. Furthermore, this region around the known TSSs is the only predicted putative CP upstream of this gene as there are no further peaks in the MAPP profile (with M_sc _>0.2) for >10,000 nucleotides. In this case, MAPP gives a very clear indication of where transcription of this gene begins.

#### *PF13_0011 (pfg27/25)*

The region incorporating the gametocyte specific gene *pfg27/25 *was chosen for analysis as the 5' region of this gene has been characterized in detail experimentally [32,35]. TSSs were identified by primer extension at -389, -394, -405 and -413 nucleotides from the ATG (black arrows in Figure 6b). Furthermore, multiple TSSs from the FULL-Malaria database are found at positions ranging from -48 to -414 nucleotides (-48, -53, -148, -151, -267, -394, -403, -411, -413, and -414 nucleotides; blue arrows in Figure 6b). The majority of transcripts (11 of 20) start in the region from -394 to -414 nucleotides, and seven of these map precisely to -413 nucleotides. The MAPP profile has a broad peak in the region from -376 to -501 nucleotides, which incorporates the principal site of agreement between the two experiments quoted above (-413 nucleotides). In fact, the multiple peaks in the -394 to -423 nucleotide region with M_sc _= 1 are in agreement with the multiple observed TSSs between these loci.

Transcripts starting from the region beyond the most upstream TSS (-414 nucleotides) were also infrequently observed in primer extension experiments (P Alano, personal communication). In these cases, primer extension and identification of large transcripts was hindered by the long unstable stretches of poly(dA) and poly(dT) in this region. The continuation of high scoring MAPP predictions between -424 and -493 nucleotides may be explained by this phenomenon.

The series of strong sharp prediction peaks further upstream are in a region with high AT content and a highly repetitive structure. The MAPP profile in this region is certainly interesting; however, practical difficulties mean that we have very little experimental data for this region and no mapped TSSs are known. While interesting, however, none of the peaks have M_sc _>0.8.

#### *PF14_0323 (pfcam)*

Previously, 47 TSSs were mapped by 5' RLM-RACE in the first 172 nucleotides upstream of the calmodulin gene (*PF14_0323*; black arrow in Figure 6c) [30]. Only 40 out of 93 transcripts were found to be correctly spliced, of which 36 originated from TSSs between the -90 and -172 nucleotides positions. On the contrary, un-spliced transcripts were shown to predominantly originate from the first 90 nucleotides upstream of the ATG codon and were shown to represent a very small fraction of the total mRNA pool.

We found that the strongest MAPP predictions overlap with the TSSs from which correctly spliced transcripts originate and that no MAPP peaks are found in the region immediately upstream of the gene start site. The MAPP profile between -150 and -200 nucleotides contains several high confidence predictions with M_sc _≥ 0.97 (151, 155, and 199 nucleotides). The MAPP profile suggests that a broad promoter is present in the region where transcription can start from several points.

Interestingly, the TSSs determined by Polson and Blackman [30] do not correspond with those present in the FULL-Malaria database (-260 and -334 nucleotides; blue arrows in Figure 6c). The MAPP profile adjacent to the TSS at -334 nucleotides indicates that a CP may be present in this region (peaks between -320 and 370 nucleotides), illustrating that MAPP predictions can help to consolidate and explain conflicting experimental data. These data suggest that several transcription start areas may be present upstream of this gene. Upstream of the illustrated region, there are no further MAPP predictions with M_sc _>0.85 for greater than 2,000 nucleotides (in the upstream region of the adjacent gene).

### MAPP as an annotation tool

We generated MAPP predictions for every nucleotide position in the *P. falciparum *genome (v2.1.4) to demonstrate some applications of MAPP on a genomic scale. In this section we discuss, on the one hand, several examples where MAPP gives us an insight into the underlying biology, and, on the other hand, some cases where the underlying biology can explain some unusual MAPP predictions. In all cases, we illustrate the forward strand prediction in green and the reverse strand prediction in black.

#### Application 1: bidirectional core promoters

A bidirectional promoter is defined as the region (shorter than 1,000 bp) between two neighboring genes that are transcribed in opposite directions. Although these promoters have been described in human and other species, little is known as yet about their role and structure. Some correlations have, however, been found between bidirectional promoter regulated genes and human diseases [36] and there is also evidence that genes under such control may be co-regulated [37]. We identified 220 bidirectional promoter regions in the genome of *P. falciparum*, with a further 249 pairs of genes separated by less than 1,500 bp. Here we present two examples with diverse TSS arrangements as predicted by MAPP. While we usually consider just the MAPP profile on the strand containing the gene of interest, here we must analyze profiles on both strands to deduce the promoter structure.

**Figure 7:**
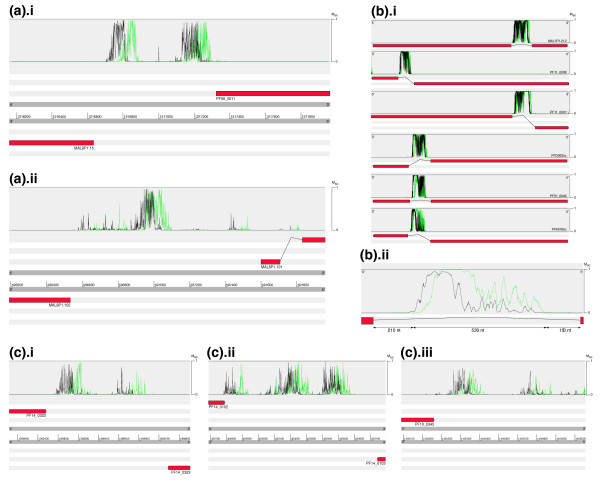
**MAPP as an annotation tool**. All figures are screenshots taken from Artemis [56]. Gene names and the M_sc _scale have been added manually and all annotated exons have been colored red. MAPP profiles in green represent forward strand predictions while those in black represent reverse strand predictions. **(a) **MAPP profiles for both DNA strands in two bidirectional promoter regions: (i) between the divergently transcribed MAL8P1.15 and *PF08_0011 *genes; (ii) between the divergently transcribed *MAL8P1.101 *and *MAL8P1.102 *genes. **(b) **(i) MAPP profiles of 6 *var *gene introns; (ii) detailed MAPP profile of the 891 nucleotides intron of *var *gene *PFA0765c*. **(c) **MAPP profiles of the 3' regions of three genes identified as having anti-sense transcripts in [45]. These MAPP predictions are on the antisense strand for each gene: (i) *PF14_0323*: *cam*; (ii) *PF14_0102*: *rap-1*; (iii) *PF10_0345*: *msp-3*.

The *MAL8P1.15 *and *PF08_0011 *genes are located on opposite DNA strands and share 692 bp 5' to their gene start sites (Figure 7a(i)). Peaks in the MAPP profiles are found just upstream of each of the gene start codons (*MAL8P1.15 *from -96 to -156 nucleotides; *PF08_0011 *from -83 to -162 nucleotides], suggesting that two distinct CPs are present in this region. As a result of MAPP prediction symmetry, we cannot establish which putative promoter is associated with which gene; however, expressed sequence tag (EST) assembly data from PlasmoDB [38] confirm that these genes are divergently transcribed. Furthermore, their transcripts arise from the regions predicted by MAPP, which suggests that the promoter for each gene is that closest to it (data not shown).

A different arrangement is found for the *MAL8P1.101 *and *MAL8P1.102 *genes (Figure 7a(ii)). Here, the MAPP profile has a single major peak in the center of the region (*MAL8P1.101 *from -558 to -585 nucleotides; *MAL8P1.102 *from -435 to -466 nucleotides) with a minor peak just upstream in the case of the forward strand. As in the previous example, EST data show that these genes are divergently transcribed, and agree with the MAPP profile. While predictions on complementary strands contribute to the promoter signal, clearly the structure of this region is such that a single CP is shared by the two genes.

These two diverse arrangements correspond with two different biological phenomena. In the first case *MAL8P1.15 *and *PF08_0011 *have similar temporal expression patterns as determined from microarray experiments [39,40], while in the second case the two genes are not co-transcribed. One may speculate that this is due to the presence of a single initiation site in the latter example, which may exclude the possibility of contemporary transcription of these two genes by separate DNA polymerase complexes. This simple example illustrates how MAPP can be used to distinguish between different architectures of *P. falciparum *bi-directional promoters. We envisage that such information may be exploited to devise novel avenues for future research on gene transcriptional control in this organism.

#### Application 2: unusual MAPP predictions illustrate biological phenomena

When screening at a genomic scale we see many unusual MAPP predictions, which is to be expected given our dearth of knowledge of regulatory regions in *P. falciparum*. We wished to assess if some of these unusual predictions were indicative of transcription-related phenomena.

The first example concerns the *var *gene introns. The multi-gene *var *family contains approximately 60 members and is of particular interest because *var *genes are regulated by a complex mechanism of allelic exclusion (only one of these genes is expressed at a time, while the rest are repressed) [41,42] involving interaction between upstream and intronic elements [43]. The MAPP profile in all *var *introns is very distinctive and is conserved among all members of the family (examples for six *var *genes are shown in Figure 7b(i)). Within the *var *intron, three different zones can be distinguished by the MAPP profile (Figure 7b(ii)). An unusually large region of high M_sc _is evident in the central part of the *var *intron occupying a region of approximately 400-600 nucleotides. The surrounding region is characterized by very low values for M_sc_. This agrees with experimental evidence that has shown that the *var *intron indeed contains a promoter in the central region [42,44], and that transcripts of different lengths arise from multiple TSSs [42].

A second example concerns genomic regions that are located downstream with respect to genes on either strand (that is, between convergently transcribed genes). These regions are not expected to contain a classical promoter; however, in many cases, strong MAPP predictions are observed. We hypothesized that such predictions may be explained by antisense transcription, a phenomenon shown to be relatively frequent in *P. falciparum *[45-47]. As antisense transcripts are transcribed by the RNA polymerase II complex in *P. falciparum *[47], it is logical to expect TSSs for antisense transcripts to be identified by MAPP even if antisense TSSs were not part of the training set. Figure 7c shows the MAPP profiles in the 3' regions of the three genes shown by Patankar *et al*. [45] to produce antisense transcripts (*PF14_0323 *(*cam*), *PF14_0102 *(*rap-1*) and *PF10_0345 *(*msp-3*)). In the case of *cam *(Figure 7c(i)) and *rap-1 *(Figure 7c(ii)) genes, the 3' region is shared with a gene on the opposite strand and strong MAPP signals are seen. For the *msp-3 *(Figure 7c(iii)) gene, there are 2,000 nucleotides between the stop and the start codons of the two adjacent genes on the same strand. Here, a MAPP peak is found approximately 150 nucleotides after the stop codon.

In addition, we examined the occurrence frequency of 3' predictions in the whole genome. We found that 40% of genes had a MAPP prediction with a M_sc _≥ 0.98 in the 1,000 nucleotides 3' to the annotated stop codon. Intriguingly, the distribution of these MAPP peaks is not random as analysis of the mean MAPP profile showed a peak between 400 and 600 nucleotides from the stop codon, similar to that observed in 5' predictions. Ultimately, the presence of strong MAPP predictions in the 3' regions suggests that such signals may indeed identify the location of TSSs for antisense transcripts.

## Conclusion

The identification of CPs in eukaryotic genomes is one of the major challenges for computational biologists due to the complex nature of promoters and high variability in their organization and sequences. This is particularly true for the highly AT rich *P. falciparum *genome. Several studies have been carried out aimed at identifying sequence elements involved in promoter activity or to specify minimal promoter elements, but to date even the most basic details of the structure of *P. falciparum *promoters have remained elusive. In this paper, we have shown that by using physicochemical and compositional properties of the DNA molecule, it is possible to train a predictor (MAPP) that identifies potential transcription start sites in the genome of *P. falciparum*. We showed that MAPP predictions strongly agree with available experimental data for well studied promoters and reveal diverse arrangements and architectures for promoter regions. Our results add to a growing body of evidence that a physicochemical code for eukaryotic genomes plays a crucial role in CP recognition.

## Materials and methods

### Datasets

We mapped 5' RACE ends for *P. falciparum *full length cDNAs [19] were mapped to the genome (v2.1) by the EST mapping algorithm Exonerate [48] using a score cutoff of 500. We mapped 9,269 out of 12,083 5'fragments to genomic locations. All 5' fragments that mapped to within 50 nucleotides of the 5' clone end were considered valid mappings. TSSs were defined as the most 5' base from each clone that mapped to the genome. Sequences that matched partially with the genomic sequences, those for which the 5' nucleotide mapped within an exon or intron, and those with duplicate TSS positions were disregarded. The region from -100 nucleotides to +49 nucleotides around each TSS was extracted, yielding a dataset of 3,553 non-redundant sequences. Sequences containing wild card characters were disregarded to leave 3,546 sequences.

Our negative dataset consisted of exonic sequences and intergenic sequences. Sequences were downloaded from PlasmoDB v5.4 [38]. The exonic dataset was generated from all *P. falciparum *exons of length longer than 150 nucleotides. For the intergenic sequence dataset, we examined the distribution of 5' untranslated region lengths (calculated from TSSs mapped previously with exonerate) and used a value above the 95th percentile (2,000 nucleotides) as a minimum distance in which TSSs are unlikely to be present. We then extracted all intergenic regions from the genome that were more than 2,000 nucleotides distant from any coding region in either direction on either strand. For both the exonic and intergenic datasets, all possible contiguous 150 nucleotide sequences were generated from each sequence. This gave 13,196 non-redundant exonic sequences and 3,407 non-redundant intergenic sequences. We randomly selected 3,407 exons from the exonic dataset and the rest were disregarded.

### Selection of physicochemical DNA properties

Values for physicochemical DNA properties were obtained from the International Centre for Genetic Engineering and Biotechnology [49,50] and from the Berkeley Drosophila Genome Project [51]. In order to eliminate redundancies, we calculated correlations between properties. To allow comparison of di- and tri-nucleotide properties, we considered the mean value of each property for all possible hexamers. Using these 6-mer values, we then calculated correlation coefficients (*r*) between all properties to generate a distance matrix (Additional data file 6). For each row (property) of this matrix, the average absolute *r *($\left|\bar{r}\right|$) was calculated. The matrix rows were then ordered by ascending $\left|\bar{r}\right|$. Starting from the first row (that with the lowest $\left|\bar{r}\right|$), we removed all those properties for which the correlation coefficient with this property was greater than 0.9. We then iterated for each row, until no further correlations greater than 0.9 were found. This resulted in the removal of 28 highly correlated properties. LD parameter tetra-nucleotide values were obtained from E Bultrini (personal communication).

### Training/test datasets

We used 1,100 CP, 2,200 EX and 2,200 IG sequence profiles to train each SVM. The sequences were all chosen at random. All sequences in the region from 3,800 bp downstream to 3,500 bp upstream of *PF13_0011*, in the region 5,000 bp upstream of *PF14_0323 *and in the region from 1,921 bp downstream to 3,000 bp upstream of *PF11_0009 *were excluded from the training set. The first test dataset (Test 1) contained 610 CP, 302 IG and 302 EX sequences and the second one (Test 2) contained 1,834 CP, 910 IG and 910 EX sequences.

### Statistical quantities

The statistical values used to assess performance throughout this paper are those used frequently in information retrieval:

$$\text{Sensitivity}=\frac{TP}{TP+FN}$$

$$\text{Specificity}=\frac{TN}{FP+TN}$$

$$\text{PPV}=\frac{TP}{TP+FP}$$

$$\text{F}=2*\frac{\left(PPV*Sen\right)}{\left(PPV+Sen\right)}$$

where TP, FP, TN and FN are the number of true positives, false positives, true negatives and false negatives, respectively.

### SVM training and feature selection

The libSVM library and additional tools were downloaded from [52,53]. For each sequence in each dataset (CP, EX and IG), 33 physicochemical property profiles were generated from the corresponding polynucleotide's property score at every nucleotide position in a sequence. Thus, each sequence of length *n *and property poly-nucleotide size, *w*, was translated into a profile vector of length (*n *- (*w *+ 1)) for each property. Profiles were then normalized to the range 0-1.

A wrapper-type feature selection algorithm was used for training 33 SVMs with radial bias function (RBF) kernels using each of the 33 sets of profiles. Each position (feature) in a set of profiles was ranked individually according to a score as described in [54] and a series of trial feature sets were generated by iteratively halving the number of top features used. This method does not account for interdependencies between features. This led to ⌊log_2 _(*n*)⌋ trial feature sets, where *n *is the total number of features. For each trial set of features, an exhaustive search for the optimal parameters of the RBF kernel was performed using fivefold cross-validation. We chose the feature combination that used the fewest features, yet was within 1% of the highest obtained accuracy. The feature selection was carried out by a modified version of the fselect.py script downloaded with the libSVM tools.

Each trained SVM outputs a score between 0 and 1. We used the outputs of the top *n *best performing SVMs {*n*|*n *∈ *Z*, 1 ≤ *n *≤ 24} as input vectors for a final RBF kernel SVM. Fivefold cross-validation was used to determine the optimal parameters for training and the -b option was used to provide probability estimates as output. A flow diagram illustrating the key points of the training and prediction processes is given in Additional data file 2.

### MAPP positional bias

We generated a prediction for every nucleotide position from - 400 to + 200 nucleotides around each TSS in the Test 2 dataset. At each position in the 601 nucleotide window, we calculated the average M_sc _(${\bar{M}}_{sc}$) for all sequences. We then counted the number of nucleotides adjacent to TSSs for which ${\bar{M}}_{sc}$ remained above the mean +1 standard deviation.

We also generated a prediction for every nucleotide position from -3,000 to +1,200 nucleotides around all *P. falciparum *gene start sites. At each position, we calculated the average M_sc _(${\bar{M}}_{sc}$) for all sequences. The TSSs distribution was generated from the 3,546 TSSs selected previously and from the distance to the closest ATG start codon for each of the FULL-Malaria flcDNA ends that mapped to coding regions.

### MAPP evaluation by EGASP criteria

Each gene with at least one TSS in the Test 2 dataset was examined. The region assessed ranged from 100 nucleotides upstream of the most 5' TSS to the gene stop codon for each gene. For each region, a MAPP score for each nucleotide was generated (MAPP profile). MAPP predictions were then clustered to allow simpler evaluation using EGASP criteria. Positive predictions were those positions that had a M_sc _≥ *t*, where *t *is the chosen threshold. Any positive predictions that were separated by ≤ 50 nucleotides were placed in the same cluster and the mean position of each cluster was then treated as a single positive prediction. At low thresholds, this method results in decreased specificity as clusters become too broad. We thus clustered predictions at all M_sc _thresholds (*t*) from 0.3 to 1 in increments of 0.1.

We performed the EGASP assessment [33] on all MAPP clustering thresholds. To evaluate true positive/false positive predictions, we used the evaluation criteria devised for the promoter prediction section of EGASP. We used window sizes (*w*) of ± 50 and ± 100 nucleotides to evaluate performance. The region examined for each gene was from *w *nucleotides upstream of the most 5' TSS to the gene stop codon. For each test sequence, true positive, false positive and false negative predictions were calculated. A prediction was considered a true positive if found within ± *w *nucleotides of a known TSS. Importantly, only one true positive prediction per TSS is counted, even if multiple predictions are present. Every prediction that fell outside TSS ± *w *nucleotide regions was considered a false positive. We calculated the PPV, sensitivity and F (harmonic mean) for each M_sc _threshold by the formulae given previously.

EP3 predictions were generated using the downloadable Java application. We performed a whole genome prediction using the Genome size <200 Mbp setting. Each site highlighted by EP3 was considered a prediction.

### Availability

Forward and reverse strand whole genome MAPP predictions are available online as gBrowse tracks in version 5.4 of PlasmoDB [38]. Flat files, genome-wide clustered predictions, SVM models and prediction scripts are available at [55] or upon request from the authors.

## Abbreviations

AUC: area under a receiver operating characteristic curve; CP: core promoter; EGASP: Encode Genome Annotation Assessment Project; EST: expressed sequence tag; EX: exonic genomic DNA; flcDNA: full-length cDNA; IG: intergenic genomic DNA; MAPP: Malaria Promoter Predictor; M_sc_: MAPP score; PPP: promoter prediction program; PPV: positive predictive value; RBF: radial bias function; SVM: support vector machine; TSS: transcription start site.

## Authors' contributions

KB carried out data analysis and computational work. JW contributed TSS data and critically evaluated the manuscript. KB and EP participated in the design and coordination of the study, the analysis of results and the writing of the manuscript. EP conceived the study. All the authors have read and approved the final version of the manuscript.

## Additional data files

The following additional data are available with the online version of this paper. Additional data file 1 is a table listing the experimentally determined DNA physicochemical properties used. Additional data file 2 provides MAPP flow diagrams illustrating the key steps in the individual SVM training/feature selection and MAPP prediction processes. Additional data file 3 provide MAPP profiles around experimentally mapped TSSs. Additional data file 4 presents MAPP predictions on both DNA strands. Additional data file 5 is the MAPP analysis around independently identified TSSs. Additional data file 6 presents correlations between DNA physicochemical properties.

## Supplementary Material

Additional data file 1 Physicochemical properties, property numbers, and data source for each. Click here for file

Additional data file 2 Key steps in the individual SVM training/feature selection and MAPP prediction processes. Click here for file

Additional data file 3 The averaged MAPP profiles in the region from -400 to +200 nucleotides around TSSs possibly highlight a transcription start area. Click here for file

Additional data file 4 Explanation of the strand symmetry and shift observed in MAPP profiles. Click here for file

Additional data file 5 The upstream regions of genes for which TSSs have been mapped experimentally and independently of the Full-Malaria database. Click here for file

Additional data file 6 These two color-coded matrices represent the correlations between physicochemical properties. On top is the matrix for all properties, while below is that for the reduced set of non-redundant properties used for the predictor. Click here for file
