# Supplementary material for: Core promoters are predicted by their distinct physicochemical properties in the genome of Plasmodium falciparum
Source: Genome Biol. 2008 Dec 18;9(12):R178. doi: 10.1186/gb-2008-9-12-r178 (PMC2646282; doi:10.1186/gb-2008-9-12-r178)
Supplement: Additional data file 1 — Physicochemical properties, property numbers, and data source for each. [file gb-2008-9-12-r178-S1.pdf]

Property #	di/tri/tetra nt	Name	Source	Reference
1	tri-nt	Bendability(DNAse)	*	[57]
2	tri-nt	Bendability(consensus)	*	[57]
3	tri-nt	Tri-nucleotide GC Content	*	[49]
4	tetra-nt	LD Parameter	**	[6]
5	tri-nt	Nucleosome positioning	*	[58]
6	tri-nt	Consensus	***	[49, 59]
7	tri-nt	Consensus_roll	***	[49, 59]
8	tri-nt	Consensus-Rigid	***	[49, 59]
9	tri-nt	DNaseI	***	[60]
10	tri-nt	DNaseI-Rigid	***	[60]
11	tri-nt	MW-Daltons	***	[49]
12	tri-nt	MW-kg	***	[49]
13	tri-nt	Nucleosome	***	[61]
14	tri-nt	Nucleosome-Rigid	***	[61]
15	di-nt	base stacking	*	[62]
16	di-nt	protein induced deformability	*	[63]
17	di-nt	B-DNA twist	*	[64]
18	di-nt	Di-nucleotide GC Content	*	[49]
19	di-nt	A-philicity	*	[65]
20	di-nt	Propeller twist	*	[66]
21	di-nt	Duplex stability (free energy)	*	[67]
22	di-nt	Duplex stability (disrupt energy)	*	[68]
23	di-nt	DNA denaturation	*	[69]
24	di-nt	Bending stiffness	*	[70]
25	di-nt	Protein-DNA twist	*	[63]
26	di-nt	Stabilising energy of Z-DNA	*	[71]
27	di-nt	Aida_BA_transition	***	[72]
28	di-nt	Bansal_roll	***	[73]
29	di-nt	Bansal_tilt	***	[73]
30	di-nt	Bansal_twist	***	[73]
31	di-nt	Bolshoy_roll	***	[74]
32	di-nt	Bolshoy_tilt	***	[74]
33	di-nt	Bolshoy_twist	***	[74]
34	di-nt	Breslauer_dG	***	[68]
35	di-nt	Breslauer_dH	***	[68]
36	di-nt	Breslauer_dS	***	[68]
37	di-nt	Calladine_roll	***	[75]
38	di-nt	DeSantis_roll	***	[76]
39	di-nt	DeSantis_tilt	***	[76]
40	di-nt	DeSantis_twist	***	[76]
41	di-nt	Electron_interaction	***	[49]
42	di-nt	Gorin_roll	***	[64]
43	di-nt	Gorin_tilt	***	[64]
44	di-nt	Gorin_twist	***	[64]
45	di-nt	Hartman_trans_free_energy	***	[77]
46	di-nt	Helix-Coil_transition	***	[78]
47	di-nt	Ivanov_BA_transition	***	[79]
48	di-nt	Lisser_BZ_transition	***	[80]
49	di-nt	Polar_interaction	***	[59]
50	di-nt	SantaLucia_dG	***	[81]
51	di-nt	SantaLucia_dH	***	[81]
52	di-nt	SantaLucia_dS	***	[81]
53	di-nt	Sarai_flexibility	***	[82]
54	di-nt	Stability	***	[83]
55	di-nt	Stacking_energy	***	[62]
56	di-nt	Sugimoto_dG	***	[67]
57	di-nt	Sugimoto_dH	***	[67]
58	di-nt	Sugimoto_dS	***	[67]
59	di-nt	Ulyanov_roll	***	[84]
60	di-nt	Ulyanov_tilt	***	[84]
61	di-nt	Ulyanov_twist	***	[84]
62	di-nt	Watson-Crick_interaction	***	[85]

* [51]
** Dr. E. Bultrini (personal communication)
*** [50]
