# Supplementary material for: Core promoters are predicted by their distinct physicochemical properties in the genome of Plasmodium falciparum
Source: Genome Biol. 2008 Dec 18;9(12):R178. doi: 10.1186/gb-2008-9-12-r178 (PMC2646282; doi:10.1186/gb-2008-9-12-r178)
Supplement: Additional data file 2 — Key steps in the individual SVM training/feature selection and MAPP prediction processes. [file gb-2008-9-12-r178-S2.pdf]

A.

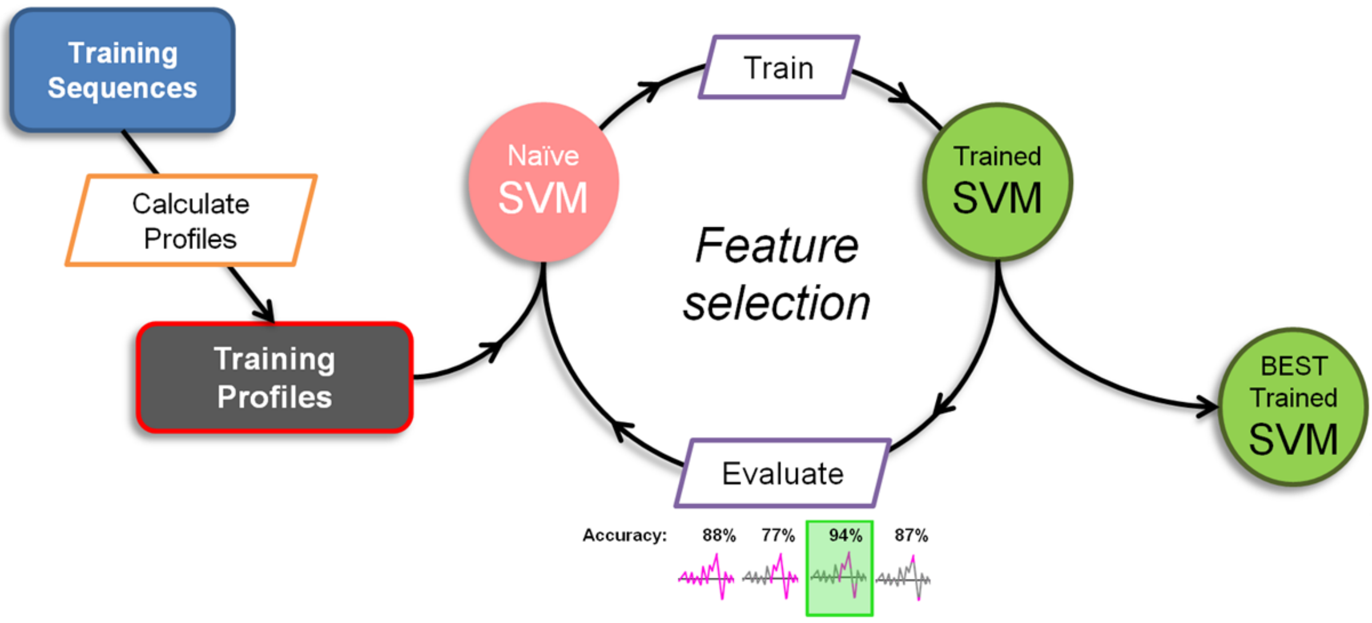

A. The training cycle for each of the single property SVMs.

Each DNA sequence is converted into a numerical physicochemical property profile and this dataset of profiles is used to train an SVM. Feature selection is an iterative cycle which trains several SVMs with different combinations of features. The accuracy of the resulting SVMs are assessed and that with the best 5-fold cross validation accuracy is used (in this example, highlighted in green). Such an SVM is trained for each of the 33 non-redundant physicochemical properties.

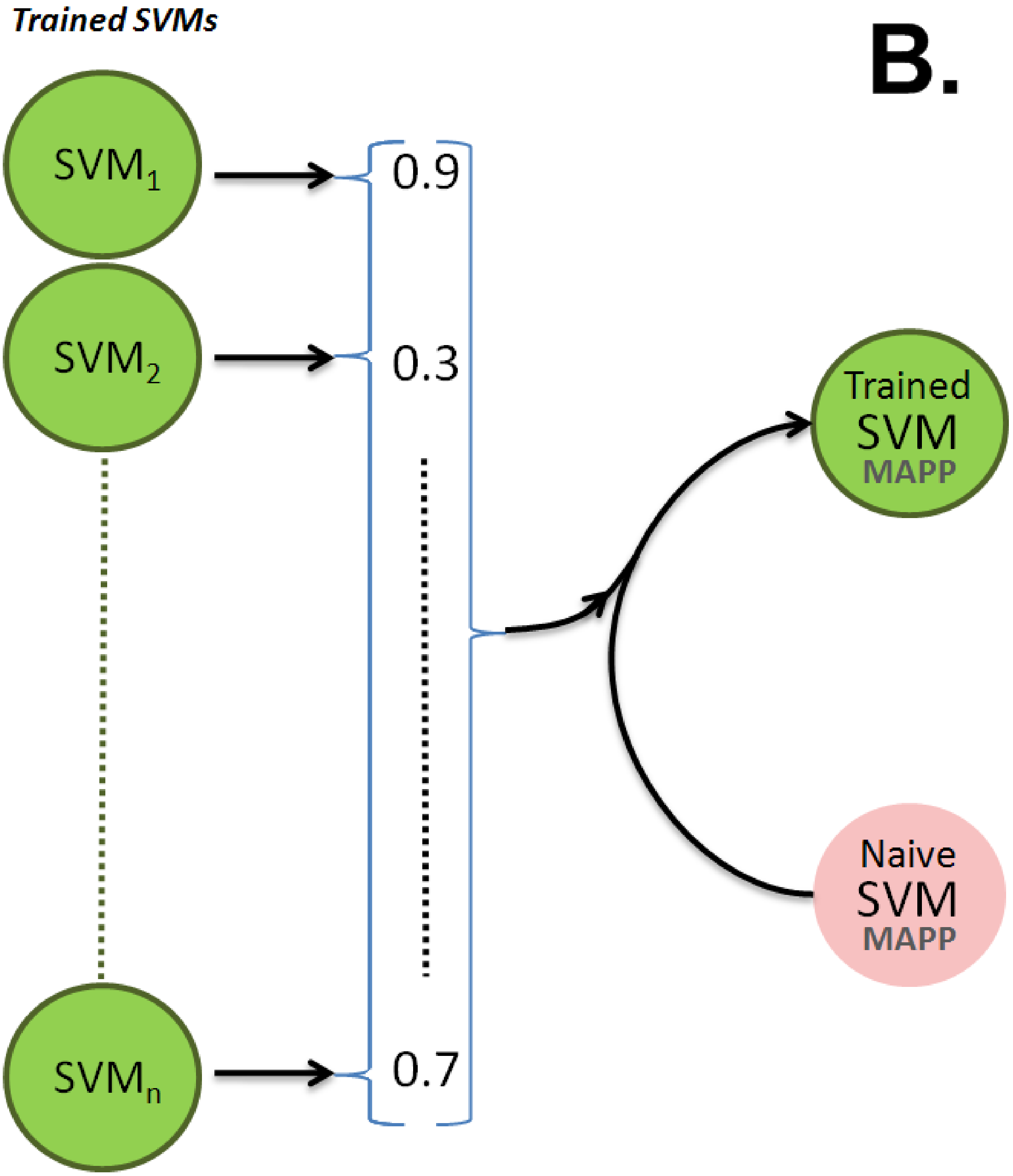

B. ***Training the combinatorial SVM***

The outputs of each of the *n* trained SVMs are combined into a single vector which is used to as the input for the final SVM (SVM_{MAPP}). Feature selection is not used for the 5-fold cross validation training process. The output is a likelihood score between 0 and 1 that the nucleotide at position 100 of the input sequence Is a TSS.

C.

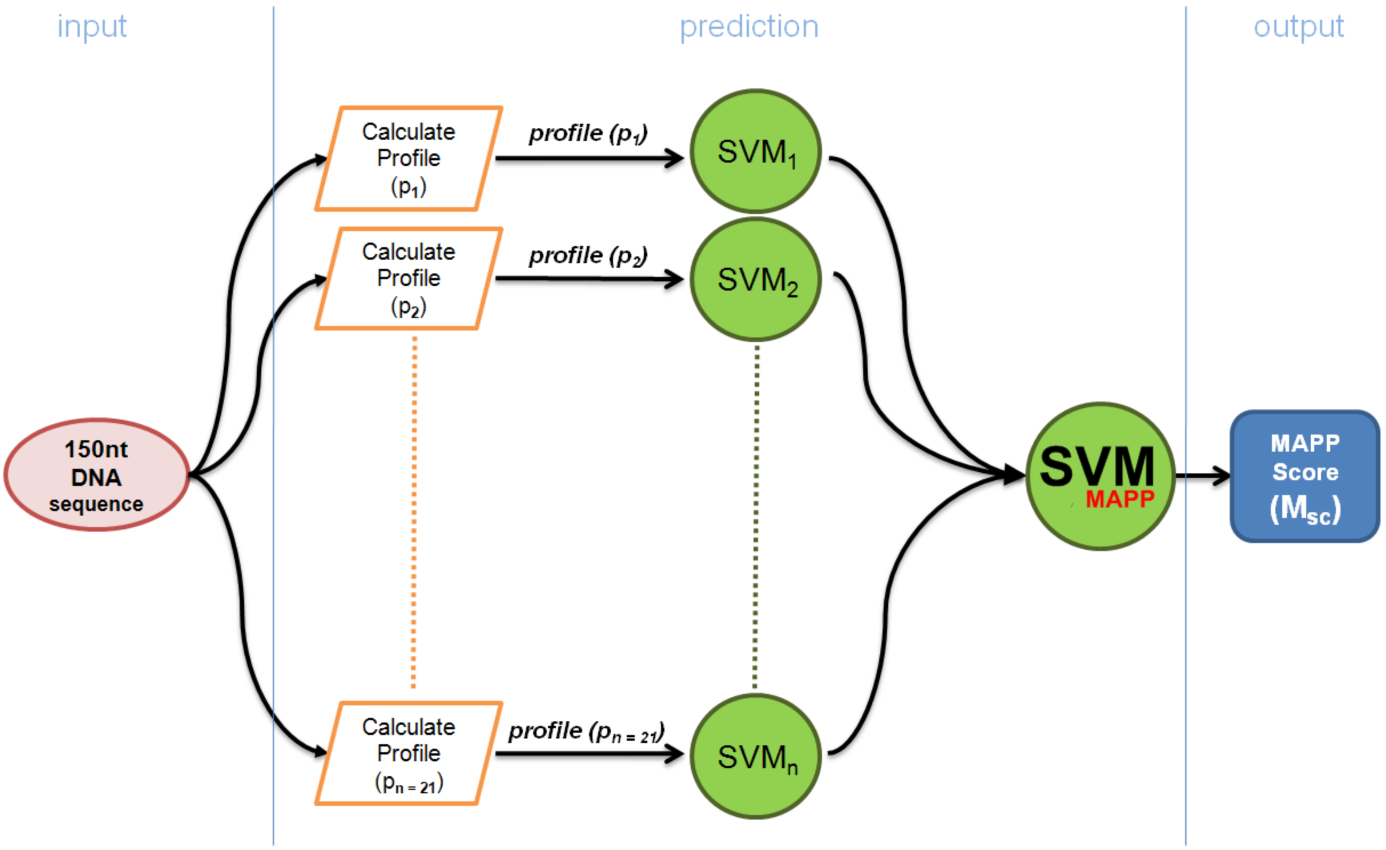

C. MAPP prediction schema.

This is a schema of how MAPP makes a TSS prediction for a given sequence. A bare 150 nt sequence is used as input. This sequence is converted into 21 physicochemical property profiles. Each profile is passed through a trained SVM (see A) for that property. The outputs of these SVMs are then combined and passed through the final SVM (SVM_{MAPP}). The output is a likelihood score that the nucleotide at position 100 of this sequence is a TSS. To generate MAPP predictions for a longer sequence, this procedure is repeated for each 150nt subsequence. Consequently, MAPP scores for the first 100 nt and the final 50 nt of a given sequence cannot be predictions.
