# Supplementary figures and images for: Core promoters are predicted by their distinct physicochemical properties in the genome of Plasmodium falciparum
Source: Genome Biol. 2008 Dec 18;9(12):R178. doi: 10.1186/gb-2008-9-12-r178 (PMC2646282; doi:10.1186/gb-2008-9-12-r178)

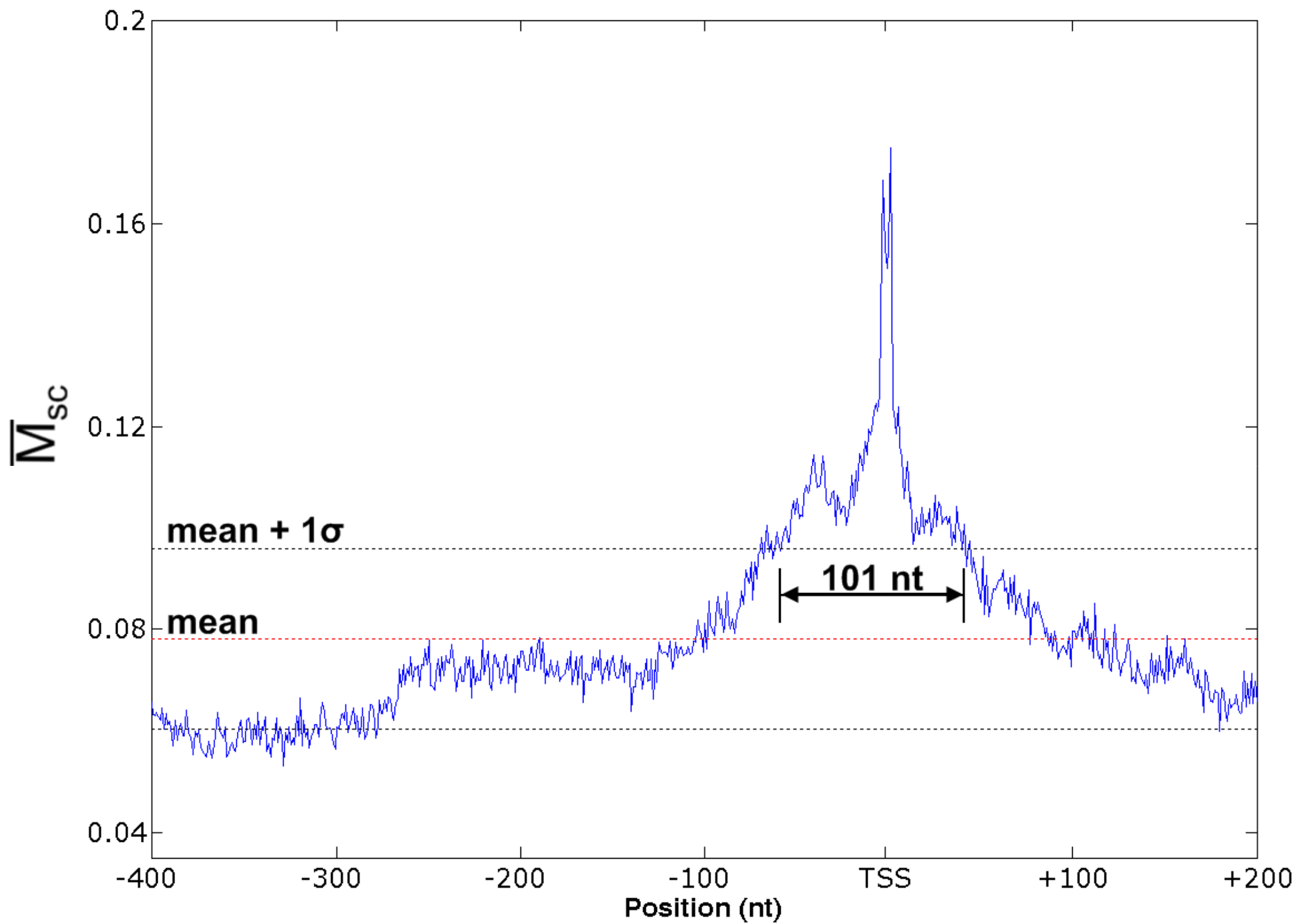

Supplement: Additional data file 3 — The averaged MAPP profiles in the region from -400 to +200 nucleotides around TSSs possibly highlight a transcription start area. [file gb-2008-9-12-r178-S3.pdf]
