# Supplementary material for: Core promoters are predicted by their distinct physicochemical properties in the genome of Plasmodium falciparum
Source: Genome Biol. 2008 Dec 18;9(12):R178. doi: 10.1186/gb-2008-9-12-r178 (PMC2646282; doi:10.1186/gb-2008-9-12-r178)

MAPP Prediction Strand Symmetry and Shift:

1. This figure illustrates the MAPP prediction mechanism moving along each of the two DNA strands. A 150 nt prediction window is shifted from 5' to 3', 1 nt at a time (here we show 10 nt at a time as a simplification). The black box represents the region of the input vector which should contain a promoter signal if the system is to output a high MAPP score (M_{sc}).

2. We expect a high M_{sc} when the TSS is at position 100 of the 150 nt window.

3. The violet region contains the strongest core promoter signals as shown in the main body of this paper. In this example, it occupies the 50 nt upstream of the only TSS.

4. The two predictions of interest are those which give a high M_{sc} (red lines).

   - on the + strand, this is a correct prediction.

   - on the - strand, the prediction will give a high M_{sc} as the strong signal upstream of the TSS falls within the important "feature zone" of the input vector.

This process results in similar, but not identical +/- strand MAPP profiles, which are shifted by between 35 and 50 nt. Furthermore, while we have marked the intermediary steps here as having low Msc, in reality, the partial overlapping of the TSS signal with the "feature rich zone" of the input vector may also explain the multiple peaks effect we see around known TSS.

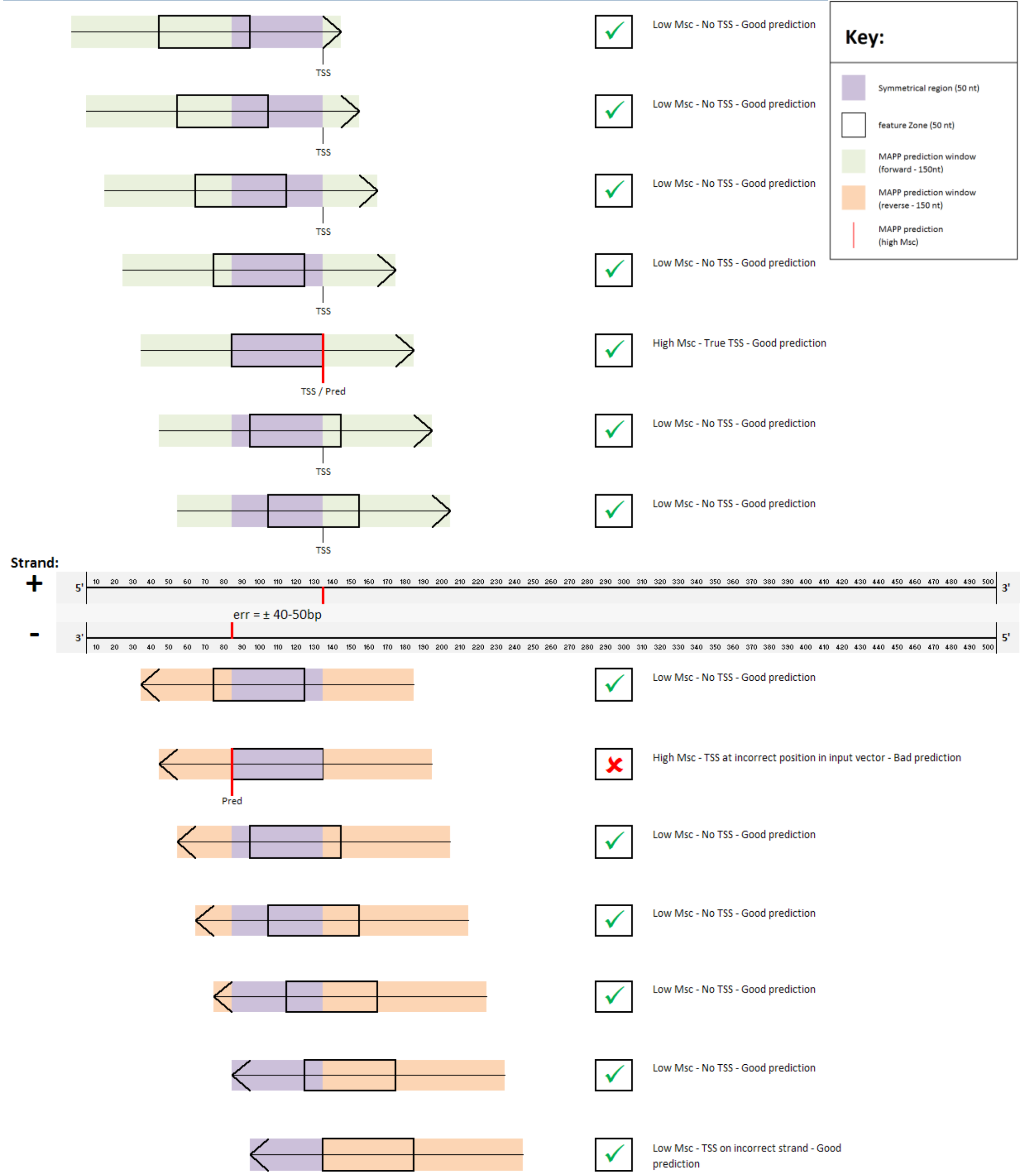

Supplement: Additional data file 4 — Explanation of the strand symmetry and shift observed in MAPP profiles. [file gb-2008-9-12-r178-S4.pdf]
