# Supplementary material for: Core promoters are predicted by their distinct physicochemical properties in the genome of Plasmodium falciparum
Source: Genome Biol. 2008 Dec 18;9(12):R178. doi: 10.1186/gb-2008-9-12-r178 (PMC2646282; doi:10.1186/gb-2008-9-12-r178)
Supplement: Additional data file 5 — The upstream regions of genes for which TSSs have been mapped experimentally and independently of the Full-Malaria database. [file gb-2008-9-12-r178-S5.pdf]

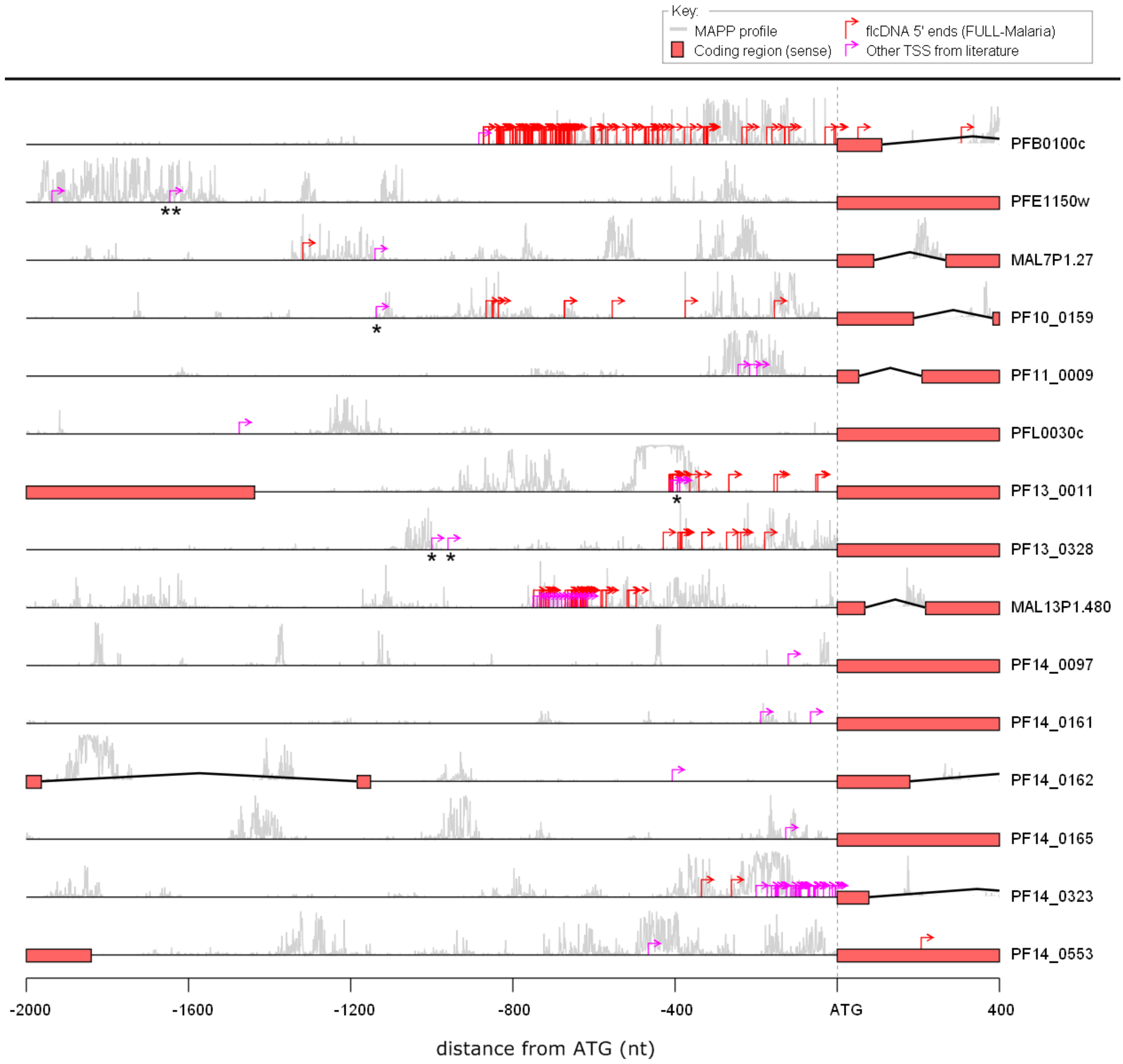

Promoter regions for which the TSS has been defined experimentally in the genome of *P. falciparum*

Independently mapped TSS are shown in pink, Full-Malaria TSSs are shown in red, while the MAPP prediction is shown as a grey profile across the region. The MAPP profile shown here is the profile on the same strand as the studied gene. In some cases (indicated with asterisks), 5’ capping methods such as RACE were not used and thus the mapped TSS may not represent the true 5’ end of the transcript. In the case of PFE1150w, TSS were identified by two different experiments. The TSS at -1647 nt represents the most 3’ end of a 669 nt region within which transcription begins (indicated with a double asterisk).
