# Supplementary material for: Core promoters are predicted by their distinct physicochemical properties in the genome of Plasmodium falciparum
Source: Genome Biol. 2008 Dec 18;9(12):R178. doi: 10.1186/gb-2008-9-12-r178 (PMC2646282; doi:10.1186/gb-2008-9-12-r178)
Supplement: Additional data file 6 — These two color-coded matrices represent the correlations between physicochemical properties. On top is the matrix for all properties, while below is that for the reduced set of non-redundant properties used for the predictor. [file gb-2008-9-12-r178-S6.pdf]

Correlations for all di/tri/tetra-nt (LD: p4) properties.  Calculated from mean scores for all 4096 possible 6-mers.

## All Properties

| | 1 | 2 | 3 | 4 | 5 | 6 | 7 | 8 | 9 | 10 | 11 | 12 | 13 | 14 | 15 | 16 | 17 | 18 | 19 | 20 | 21 | 22 | 23 | 24 | 25 | 26 | 27 | 28 |
|---|---|---|---|---|---|---|---|---|---|---|---|---|---|---|---|---|---|---|---|---|---|---|---|---|---|---|---|---|
| 1 | 1.00 | 0.82 | 0.14 | 0.14 | 0.37 | 0.82 | 0.82 | 0.82 | 0.37 | 1.00 | 0.12 | 0.12 | 0.37 | 0.37 | 0.14 | 0.15 | 0.07 | 0.14 | 0.06 | 0.36 | 0.02 | 0.10 | 0.08 | 0.02 | 0.01 | 0.15 | 0.15 | 0.22 |
| 2 | 0.82 | 1.00 | 0.60 | 0.06 | 0.84 | 1.00 | 1.00 | 1.00 | 0.54 | 0.82 | 0.57 | 0.57 | 0.83 | 0.84 | 0.60 | 0.48 | 0.03 | 0.59 | 0.28 | 0.69 | 0.49 | 0.33 | 0.54 | 0.44 | 0.08 | 0.48 | 0.49 | 0.46 |
| 3 | 0.14 | 0.60 | 1.00 | 0.06 | 0.84 | 0.60 | 0.60 | 0.60 | 0.43 | 0.15 | 0.94 | 0.94 | 0.84 | 0.84 | 0.86 | 0.69 | 0.19 | 0.98 | 0.60 | 0.86 | 0.95 | 0.86 | 0.91 | 0.93 | 0.16 | 0.80 | 0.54 | 0.61 |
| 4 | 0.14 | 0.06 | 0.06 | 1.00 | 0.03 | 0.06 | 0.06 | 0.06 | 0.22 | 0.13 | 0.06 | 0.06 | 0.03 | 0.03 | 0.06 | 0.01 | 0.06 | 0.06 | 0.01 | 0.01 | 0.05 | 0.04 | 0.06 | 0.06 | 0.03 | 0.05 | 0.03 | 0.06 |
| 5 | 0.37 | 0.84 | 0.84 | 0.03 | 1.00 | 0.83 | 0.83 | 0.83 | 0.52 | 0.38 | 0.81 | 0.81 | 1.00 | 1.00 | 0.83 | 0.63 | 0.02 | 0.83 | 0.40 | 0.77 | 0.78 | 0.63 | 0.80 | 0.70 | 0.13 | 0.64 | 0.65 | 0.55 |
| 6 | 0.82 | 1.00 | 0.60 | 0.06 | 0.83 | 1.00 | 1.00 | 1.00 | 0.54 | 0.83 | 0.57 | 0.57 | 0.83 | 0.84 | 0.60 | 0.48 | 0.03 | 0.59 | 0.28 | 0.69 | 0.49 | 0.33 | 0.53 | 0.44 | 0.08 | 0.48 | 0.49 | 0.46 |
| 7 | 0.82 | 1.00 | 0.60 | 0.06 | 0.83 | 1.00 | 1.00 | 1.00 | 0.54 | 0.83 | 0.57 | 0.57 | 0.83 | 0.84 | 0.60 | 0.48 | 0.03 | 0.59 | 0.28 | 0.69 | 0.49 | 0.33 | 0.53 | 0.44 | 0.08 | 0.48 | 0.49 | 0.46 |
| 8 | 0.82 | 1.00 | 0.60 | 0.06 | 0.83 | 1.00 | 1.00 | 1.00 | 0.53 | 0.83 | 0.57 | 0.57 | 0.83 | 0.84 | 0.60 | 0.48 | 0.02 | 0.59 | 0.29 | 0.69 | 0.49 | 0.33 | 0.54 | 0.44 | 0.08 | 0.48 | 0.49 | 0.46 |
| 9 | 0.37 | 0.54 | 0.43 | 0.22 | 0.52 | 0.54 | 0.54 | 0.53 | 1.00 | 0.37 | 0.39 | 0.39 | 0.52 | 0.52 | 0.40 | 0.29 | 0.11 | 0.41 | 0.24 | 0.41 | 0.39 | 0.35 | 0.43 | 0.33 | 0.09 | 0.33 | 0.30 | 0.37 |
| 10 | 1.00 | 0.82 | 0.15 | 0.13 | 0.38 | 0.83 | 0.83 | 0.83 | 0.37 | 1.00 | 0.13 | 0.13 | 0.38 | 0.38 | 0.15 | 0.15 | 0.06 | 0.15 | 0.07 | 0.37 | 0.03 | 0.09 | 0.09 | 0.02 | 0.01 | 0.15 | 0.15 | 0.22 |
| 11 | 0.12 | 0.57 | 0.94 | 0.06 | 0.81 | 0.57 | 0.57 | 0.57 | 0.39 | 0.13 | 1.00 | 1.00 | 0.81 | 0.81 | 0.82 | 0.66 | 0.18 | 0.94 | 0.58 | 0.82 | 0.92 | 0.82 | 0.87 | 0.90 | 0.16 | 0.77 | 0.52 | 0.58 |
| 12 | 0.12 | 0.57 | 0.94 | 0.06 | 0.81 | 0.57 | 0.57 | 0.57 | 0.39 | 0.13 | 1.00 | 1.00 | 0.81 | 0.81 | 0.82 | 0.66 | 0.18 | 0.94 | 0.58 | 0.82 | 0.92 | 0.82 | 0.87 | 0.90 | 0.16 | 0.77 | 0.52 | 0.58 |
| 13 | 0.37 | 0.83 | 0.84 | 0.03 | 1.00 | 0.83 | 0.83 | 0.83 | 0.52 | 0.38 | 0.81 | 0.81 | 1.00 | 1.00 | 0.83 | 0.63 | 0.02 | 0.82 | 0.40 | 0.77 | 0.78 | 0.63 | 0.79 | 0.70 | 0.13 | 0.64 | 0.65 | 0.54 |
| 14 | 0.37 | 0.84 | 0.84 | 0.03 | 1.00 | 0.84 | 0.84 | 0.84 | 0.52 | 0.38 | 0.81 | 0.81 | 1.00 | 1.00 | 0.83 | 0.63 | 0.02 | 0.82 | 0.40 | 0.77 | 0.78 | 0.63 | 0.79 | 0.70 | 0.13 | 0.64 | 0.65 | 0.54 |
| 15 | 0.14 | 0.60 | 0.86 | 0.06 | 0.83 | 0.60 | 0.60 | 0.60 | 0.40 | 0.15 | 0.82 | 0.82 | 0.83 | 0.83 | 1.00 | 0.62 | 0.08 | 0.87 | 0.40 | 0.68 | 0.86 | 0.71 | 0.92 | 0.72 | 0.07 | 0.60 | 0.74 | 0.41 |
| 16 | 0.15 | 0.48 | 0.69 | 0.01 | 0.63 | 0.48 | 0.48 | 0.48 | 0.29 | 0.15 | 0.66 | 0.66 | 0.63 | 0.63 | 0.62 | 1.00 | 0.08 | 0.70 | 0.20 | 0.76 | 0.77 | 0.70 | 0.52 | 0.61 | 0.33 | 0.87 | 0.78 | 0.36 |
| 17 | 0.07 | 0.03 | 0.19 | 0.06 | 0.02 | 0.03 | 0.03 | 0.02 | 0.11 | 0.06 | 0.18 | 0.18 | 0.02 | 0.02 | 0.08 | 0.08 | 1.00 | 0.20 | 0.62 | 0.08 | 0.18 | 0.18 | 0.30 | 0.18 | 0.11 | 0.24 | 0.26 | 0.12 |
| 18 | 0.14 | 0.59 | 0.98 | 0.06 | 0.83 | 0.59 | 0.59 | 0.59 | 0.41 | 0.15 | 0.94 | 0.94 | 0.82 | 0.82 | 0.87 | 0.70 | 0.20 | 1.00 | 0.61 | 0.87 | 0.97 | 0.87 | 0.93 | 0.95 | 0.17 | 0.82 | 0.56 | 0.62 |
| 19 | 0.06 | 0.28 | 0.60 | 0.01 | 0.40 | 0.28 | 0.28 | 0.29 | 0.24 | 0.07 | 0.58 | 0.58 | 0.40 | 0.40 | 0.40 | 0.20 | 0.62 | 0.61 | 1.00 | 0.63 | 0.52 | 0.44 | 0.64 | 0.66 | 0.62 | 0.38 | 0.06 | 0.23 |
| 20 | 0.36 | 0.69 | 0.86 | 0.01 | 0.77 | 0.69 | 0.69 | 0.69 | 0.41 | 0.37 | 0.82 | 0.82 | 0.77 | 0.77 | 0.68 | 0.76 | 0.08 | 0.87 | 0.63 | 1.00 | 0.81 | 0.68 | 0.74 | 0.84 | 0.20 | 0.79 | 0.53 | 0.56 |
| 21 | 0.02 | 0.49 | 0.95 | 0.05 | 0.78 | 0.49 | 0.49 | 0.49 | 0.39 | 0.03 | 0.92 | 0.92 | 0.78 | 0.78 | 0.86 | 0.77 | 0.18 | 0.97 | 0.52 | 0.81 | 1.00 | 0.94 | 0.90 | 0.92 | 0.06 | 0.84 | 0.63 | 0.58 |
| 22 | 0.10 | 0.33 | 0.86 | 0.04 | 0.63 | 0.33 | 0.33 | 0.33 | 0.35 | 0.09 | 0.82 | 0.82 | 0.63 | 0.63 | 0.71 | 0.70 | 0.18 | 0.87 | 0.44 | 0.68 | 0.94 | 1.00 | 0.80 | 0.87 | 0.02 | 0.78 | 0.53 | 0.56 |
| 23 | 0.08 | 0.54 | 0.91 | 0.06 | 0.80 | 0.53 | 0.53 | 0.54 | 0.43 | 0.09 | 0.87 | 0.87 | 0.79 | 0.79 | 0.92 | 0.52 | 0.30 | 0.93 | 0.64 | 0.74 | 0.90 | 0.80 | 1.00 | 0.84 | 0.29 | 0.62 | 0.55 | 0.57 |
| 24 | 0.02 | 0.44 | 0.93 | 0.06 | 0.70 | 0.44 | 0.44 | 0.44 | 0.33 | 0.02 | 0.90 | 0.90 | 0.70 | 0.70 | 0.72 | 0.61 | 0.18 | 0.95 | 0.66 | 0.84 | 0.92 | 0.87 | 0.84 | 1.00 | 0.24 | 0.78 | 0.35 | 0.62 |
| 25 | 0.01 | 0.08 | 0.16 | 0.03 | 0.13 | 0.08 | 0.08 | 0.08 | 0.09 | 0.01 | 0.16 | 0.16 | 0.13 | 0.13 | 0.07 | 0.33 | 0.11 | 0.17 | 0.62 | 0.20 | 0.06 | 0.02 | 0.29 | 0.24 | 1.00 | 0.28 | 0.12 | 0.11 |
| 26 | 0.15 | 0.48 | 0.80 | 0.05 | 0.64 | 0.48 | 0.48 | 0.48 | 0.33 | 0.15 | 0.77 | 0.77 | 0.64 | 0.64 | 0.60 | 0.87 | 0.24 | 0.82 | 0.38 | 0.79 | 0.84 | 0.78 | 0.62 | 0.78 | 0.28 | 1.00 | 0.47 | 0.61 |
| 27 | 0.15 | 0.49 | 0.54 | 0.03 | 0.65 | 0.49 | 0.49 | 0.49 | 0.30 | 0.15 | 0.52 | 0.52 | 0.65 | 0.65 | 0.74 | 0.78 | 0.26 | 0.56 | 0.06 | 0.53 | 0.63 | 0.53 | 0.55 | 0.35 | 0.12 | 0.47 | 1.00 | 0.13 |
| 28 | 0.22 | 0.46 | 0.61 | 0.06 | 0.55 | 0.46 | 0.46 | 0.46 | 0.37 | 0.22 | 0.58 | 0.58 | 0.54 | 0.54 | 0.41 | 0.36 | 0.12 | 0.62 | 0.23 | 0.56 | 0.58 | 0.56 | 0.57 | 0.62 | 0.11 | 0.61 | 0.13 | 1.00 |
| 29 | 0.47 | 0.42 | 0.06 | 0.07 | 0.22 | 0.42 | 0.42 | 0.42 | 0.19 | 0.47 | 0.06 | 0.06 | 0.22 | 0.23 | 0.22 | 0.17 | 0.09 | 0.06 | 0.01 | 0.10 | 0.10 | 0.25 | 0.06 | 0.33 | 0.07 | 0.06 | 0.50 | 0.23 |
| 30 | 0.22 | 0.20 | 0.12 | 0.04 | 0.11 | 0.20 | 0.20 | 0.19 | 0.02 | 0.22 | 0.11 | 0.11 | 0.11 | 0.11 | 0.03 | 0.42 | 0.56 | 0.12 | 0.59 | 0.09 | 0.08 | 0.15 | 0.18 | 0.21 | 0.58 | 0.16 | 0.40 | 0.12 |
| 31 | 0.21 | 0.20 | 0.11 | 0.09 | 0.13 | 0.21 | 0.21 | 0.21 | 0.01 | 0.21 | 0.11 | 0.11 | 0.13 | 0.14 | 0.03 | 0.35 | 0.25 | 0.12 | 0.31 | 0.50 | 0.05 | 0.07 | 0.02 | 0.19 | 0.14 | 0.14 | 0.17 | 0.16 |
| 32 | 0.29 | 0.33 | 0.22 | 0.08 | 0.26 | 0.33 | 0.33 | 0.33 | 0.33 | 0.29 | 0.22 | 0.22 | 0.26 | 0.26 | 0.20 | 0.13 | 0.50 | 0.23 | 0.55 | 0.34 | 0.21 | 0.24 | 0.36 | 0.11 | 0.40 | 0.10 | 0.28 | 0.13 |
| 33 | 0.14 | 0.12 | 0.10 | 0.03 | 0.05 | 0.12 | 0.12 | 0.12 | 0.03 | 0.15 | 0.10 | 0.10 | 0.05 | 0.05 | 0.09 | 0.17 | 0.45 | 0.10 | 0.46 | 0.10 | 0.01 | 0.09 | 0.02 | 0.09 | 0.46 | 0.07 | 0.23 | 0.10 |
| 34 | 0.11 | 0.33 | 0.85 | 0.03 | 0.65 | 0.33 | 0.33 | 0.33 | 0.33 | 0.11 | 0.81 | 0.81 | 0.65 | 0.64 | 0.72 | 0.71 | 0.02 | 0.86 | 0.37 | 0.68 | 0.94 | 0.98 | 0.78 | 0.86 | 0.05 | 0.74 | 0.59 | 0.55 |
| 35 | 0.26 | 0.09 | 0.62 | 0.01 | 0.39 | 0.09 | 0.09 | 0.08 | 0.26 | 0.25 | 0.60 | 0.60 | 0.39 | 0.39 | 0.47 | 0.45 | 0.13 | 0.64 | 0.37 | 0.43 | 0.74 | 0.90 | 0.62 | 0.68 | 0.18 | 0.48 | 0.39 | 0.40 |
| 36 | 0.32 | 0.06 | 0.43 | 0.01 | 0.20 | 0.06 | 0.06 | 0.07 | 0.19 | 0.32 | 0.42 | 0.42 | 0.20 | 0.20 | 0.28 | 0.25 | 0.17 | 0.44 | 0.34 | 0.24 | 0.56 | 0.77 | 0.47 | 0.51 | 0.25 | 0.28 | 0.24 | 0.27 |
| 37 | 0.64 | 0.60 | 0.28 | 0.08 | 0.36 | 0.61 | 0.61 | 0.61 | 0.29 | 0.65 | 0.27 | 0.27 | 0.36 | 0.37 | 0.21 | 0.24 | 0.21 | 0.28 | 0.52 | 0.57 | 0.14 | 0.02 | 0.23 | 0.17 | 0.25 | 0.22 | 0.21 | 0.02 |
| 38 | 0.02 | 0.29 | 0.70 | 0.13 | 0.48 | 0.28 | 0.28 | 0.29 | 0.19 | 0.00 | 0.67 | 0.67 | 0.48 | 0.48 | 0.57 | 0.10 | 0.41 | 0.71 | 0.63 | 0.46 | 0.61 | 0.49 | 0.67 | 0.74 | 0.32 | 0.45 | 0.05 | 0.41 |
| 39 | 0.01 | 0.00 | 0.03 | 0.10 | 0.01 | 0.00 | 0.00 | 0.01 | 0.11 | 0.00 | 0.03 | 0.03 | 0.01 | 0.02 | 0.12 | 0.27 | 0.41 | 0.03 | 0.33 | 0.05 | 0.09 | 0.33 | 0.10 | 0.02 | 0.15 | 0.07 | 0.28 | 0.17 |
| 40 | 0.15 | 0.50 | 0.82 | 0.03 | 0.68 | 0.50 | 0.50 | 0.50 | 0.40 | 0.15 | 0.79 | 0.79 | 0.68 | 0.68 | 0.64 | 0.74 | 0.13 | 0.83 | 0.65 | 0.90 | 0.84 | 0.83 | 0.78 | 0.83 | 0.25 | 0.72 | 0.57 | 0.52 |
| 41 | 0.14 | 0.59 | 0.98 | 0.06 | 0.83 | 0.59 | 0.59 | 0.59 | 0.41 | 0.15 | 0.94 | 0.94 | 0.82 | 0.82 | 0.87 | 0.70 | 0.20 | 1.00 | 0.61 | 0.87 | 0.97 | 0.87 | 0.93 | 0.95 | 0.17 | 0.82 | 0.56 | 0.62 |
| 42 | 0.09 | 0.46 | 0.84 | 0.01 | 0.65 | 0.45 | 0.45 | 0.45 | 0.43 | 0.10 | 0.81 | 0.81 | 0.65 | 0.65 | 0.65 | 0.58 | 0.28 | 0.86 | 0.60 | 0.75 | 0.87 | 0.93 | 0.81 | 0.84 | 0.23 | 0.70 | 0.44 | 0.60 |
| 43 | 0.15 | 0.17 | 0.23 | 0.10 | 0.13 | 0.16 | 0.16 | 0.17 | 0.10 | 0.15 | 0.22 | 0.22 | 0.13 | 0.13 | 0.03 | 0.32 | 0.52 | 0.23 | 0.44 | 0.14 | 0.08 | 0.02 | 0.23 | 0.29 | 0.38 | 0.13 | 0.52 | 0.50 |
| 44 | 0.14 | 0.13 | 0.57 | 0.06 | 0.35 | 0.13 | 0.13 | 0.13 | 0.19 | 0.14 | 0.54 | 0.54 | 0.35 | 0.35 | 0.32 | 0.16 | 0.44 | 0.58 | 0.63 | 0.39 | 0.57 | 0.57 | 0.52 | 0.62 | 0.53 | 0.41 | 0.06 | 0.49 |
| 45 | 0.01 | 0.44 | 0.79 | 0.06 | 0.72 | 0.44 | 0.44 | 0.44 | 0.29 | 0.01 | 0.76 | 0.76 | 0.71 | 0.72 | 0.88 | 0.36 | 0.02 | 0.80 | 0.52 | 0.61 | 0.75 | 0.60 | 0.88 | 0.75 | 0.41 | 0.37 | 0.52 | 0.35 |
| 46 | 0.11 | 0.33 | 0.85 | 0.03 | 0.65 | 0.33 | 0.33 | 0.33 | 0.33 | 0.10 | 0.81 | 0.81 | 0.65 | 0.64 | 0.72 | 0.71 | 0.02 | 0.86 | 0.37 | 0.68 | 0.94 | 0.98 | 0.78 | 0.86 | 0.05 | 0.74 | 0.59 | 0.55 |
| 47 | 0.06 | 0.28 | 0.60 | 0.01 | 0.40 | 0.28 | 0.28 | 0.29 | 0.24 | 0.07 | 0.58 | 0.58 | 0.40 | 0.40 | 0.40 | 0.20 | 0.62 | 0.61 | 1.00 | 0.63 | 0.52 | 0.44 | 0.64 | 0.66 | 0.62 | 0.38 | 0.06 | 0.23 |
| 48 | 0.00 | 0.44 | 0.79 | 0.06 | 0.72 | 0.44 | 0.44 | 0.44 | 0.29 | 0.01 | 0.76 | 0.76 | 0.72 | 0.72 | 0.88 | 0.36 | 0.02 | 0.80 | 0.52 | 0.61 | 0.76 | 0.60 | 0.88 | 0.76 | 0.42 | 0.37 | 0.52 | 0.35 |
| 49 | 0.14 | 0.59 | 0.98 | 0.06 | 0.83 | 0.59 | 0.59 | 0.59 | 0.41 | 0.15 | 0.94 | 0.94 | 0.82 | 0.82 | 0.87 | 0.70 | 0.20 | 1.00 | 0.61 | 0.87 | 0.97 | 0.87 | 0.93 | 0.95 | 0.17 | 0.82 | 0.56 | 0.62 |
| 50 | 0.03 | 0.52 | 0.94 | 0.07 | 0.81 | 0.52 | 0.52 | 0.52 | 0.39 | 0.04 | 0.91 | 0.91 | 0.81 | 0.81 | 0.93 | 0.74 | 0.12 | 0.96 | 0.44 | 0.76 | 0.98 | 0.90 | 0.93 | 0.88 | 0.02 | 0.79 | 0.67 | 0.57 |
| 51 | 0.21 | 0.14 | 0.42 | 0.04 | 0.42 | 0.14 | 0.14 | 0.14 | 0.21 | 0.20 | 0.41 | 0.41 | 0.43 | 0.42 | 0.68 | 0.53 | 0.07 | 0.43 | 0.05 | 0.17 | 0.57 | 0.57 | 0.57 | 0.26 | 0.32 | 0.38 | 0.74 | 0.13 |
| 52 | 0.26 | 0.09 | 0.02 | 0.01 | 0.10 | 0.09 | 0.09 | 0.09 | 0.06 | 0.26 | 0.02 | 0.02 | 0.11 | 0.10 | 0.36 | 0.26 | 0.01 | 0.02 | 0.31 | 0.20 | 0.18 | 0.22 | 0.21 | 0.16 | 0.42 | 0.03 | 0.58 | 0.16 |
| 53 | 0.25 | 0.01 | 0.51 | 0.15 | 0.22 | 0.02 | 0.02 | 0.01 | 0.10 | 0.25 | 0.49 | 0.49 | 0.22 | 0.22 | 0.33 | 0.13 | 0.47 | 0.52 | 0.26 | 0.18 | 0.53 | 0.58 | 0.46 | 0.60 | 0.15 | 0.53 | 0.20 | 0.51 |
| 54 | 0.07 | 0.54 | 0.89 | 0.06 | 0.81 | 0.54 | 0.54 | 0.54 | 0.40 | 0.08 | 0.86 | 0.86 | 0.81 | 0.81 | 0.97 | 0.58 | 0.11 | 0.91 | 0.49 | 0.72 | 0.90 | 0.78 | 0.97 | 0.82 | 0.16 | 0.61 | 0.64 | 0.48 |
| 55 | 0.14 | 0.60 | 0.86 | 0.06 | 0.83 | 0.59 | 0.59 | 0.59 | 0.40 | 0.15 | 0.82 | 0.82 | 0.83 | 0.83 | 1.00 | 0.62 | 0.08 | 0.87 | 0.40 | 0.68 | 0.86 | 0.71 | 0.92 | 0.72 | 0.07 | 0.61 | 0.74 | 0.41 |
| 56 | 0.03 | 0.51 | 0.94 | 0.06 | 0.79 | 0.50 | 0.50 | 0.50 | 0.39 | 0.04 | 0.91 | 0.91 | 0.79 | 0.79 | 0.89 | 0.78 | 0.18 | 0.96 | 0.49 | 0.78 | 1.00 | 0.93 | 0.91 | 0.89 | 0.02 | 0.84 | 0.67 | 0.57 |
| 57 | 0.15 | 0.32 | 0.87 | 0.05 | 0.66 | 0.31 | 0.31 | 0.32 | 0.28 | 0.14 | 0.84 | 0.84 | 0.66 | 0.66 | 0.81 | 0.74 | 0.20 | 0.89 | 0.53 | 0.72 | 0.95 | 0.90 | 0.85 | 0.88 | 0.00 | 0.78 | 0.59 | 0.38 |
| 58 | 0.22 | 0.23 | 0.81 | 0.05 | 0.58 | 0.23 | 0.23 | 0.23 | 0.22 | 0.21 | 0.78 | 0.78 | 0.58 | 0.58 | 0.75 | 0.71 | 0.20 | 0.83 | 0.53 | 0.67 | 0.89 | 0.86 | 0.79 | 0.84 | 0.00 | 0.72 | 0.54 | 0.28 |
| 59 | 0.30 | 0.62 | 0.71 | 0.00 | 0.73 | 0.62 | 0.62 | 0.62 | 0.37 | 0.30 | 0.69 | 0.69 | 0.73 | 0.73 | 0.63 | 0.91 | 0.20 | 0.73 | 0.13 | 0.81 | 0.75 | 0.65 | 0.57 | 0.63 | 0.20 | 0.84 | 0.73 | 0.63 |
| 60 | 0.02 | 0.04 | 0.25 | 0.11 | 0.08 | 0.03 | 0.03 | 0.03 | 0.04 | 0.02 | 0.24 | 0.24 | 0.08 | 0.08 | 0.08 | 0.21 | 0.06 | 0.25 | 0.05 | 0.05 | 0.16 | 0.13 | 0.09 | 0.37 | 0.09 | 0.12 | 0.40 | 0.25 |
| 61 | 0.08 | 0.04 | 0.02 | 0.01 | 0.02 | 0.04 | 0.04 | 0.03 | 0.10 | 0.08 | 0.02 | 0.02 | 0.02 | 0.01 | 0.26 | 0.09 | 0.54 | 0.02 | 0.28 | 0.14 | 0.07 | 0.10 | 0.26 | 0.13 | 0.02 | 0.14 | 0.21 | 0.40 |
| 62 | 0.14 | 0.59 | 0.98 | 0.06 | 0.83 | 0.59 | 0.59 | 0.59 | 0.41 | 0.15 | 0.94 | 0.94 | 0.82 | 0.82 | 0.87 | 0.70 | 0.20 | 1.00 | 0.61 | 0.87 | 0.97 | 0.87 | 0.93 | 0.95 | 0.17 | 0.82 | 0.56 | 0.62 |

| | 29 | 30 | 31 | 32 | 33 | 34 | 35 | 36 | 37 | 38 | 39 | 40 | 41 | 42 | 43 | 44 | 45 | 46 | 47 | 48 | 49 | 50 | 51 | 52 | 53 | 54 | 55 | 56 | 57 | 58 | 59 | 60 | 61 | 62 |
|---|---|---|---|---|---|---|---|---|---|---|---|---|---|---|---|---|---|---|---|---|---|---|---|---|---|---|---|---|---|---|---|---|---|---|
| 1 | 0.47 | 0.22 | 0.21 | 0.29 | 0.14 | 0.11 | 0.26 | 0.32 | 0.64 | 0.02 | 0.01 | 0.15 | 0.14 | 0.09 | 0.15 | 0.14 | 0.01 | 0.11 | 0.06 | 0.00 | 0.14 | 0.03 | 0.21 | 0.26 | 0.25 | 0.07 | 0.14 | 0.03 | 0.15 | 0.22 | 0.30 | 0.02 | 0.08 | 0.14 |
| 2 | 0.42 | 0.20 | 0.20 | 0.33 | 0.12 | 0.33 | 0.09 | 0.06 | 0.60 | 0.29 | 0.00 | 0.50 | 0.59 | 0.46 | 0.17 | 0.13 | 0.44 | 0.33 | 0.28 | 0.44 | 0.59 | 0.52 | 0.14 | 0.09 | 0.01 | 0.54 | 0.60 | 0.51 | 0.32 | 0.23 | 0.62 | 0.04 | 0.04 | 0.59 |
| 3 | 0.06 | 0.12 | 0.11 | 0.22 | 0.10 | 0.85 | 0.62 | 0.43 | 0.28 | 0.70 | 0.03 | 0.82 | 0.98 | 0.84 | 0.23 | 0.57 | 0.79 | 0.85 | 0.60 | 0.79 | 0.98 | 0.94 | 0.42 | 0.02 | 0.51 | 0.89 | 0.86 | 0.94 | 0.87 | 0.81 | 0.71 | 0.25 | 0.02 | 0.98 |
| 4 | 0.07 | 0.04 | 0.09 | 0.08 | 0.03 | 0.03 | 0.01 | 0.01 | 0.08 | 0.13 | 0.10 | 0.03 | 0.06 | 0.01 | 0.10 | 0.06 | 0.06 | 0.03 | 0.01 | 0.06 | 0.06 | 0.07 | 0.04 | 0.01 | 0.15 | 0.06 | 0.06 | 0.06 | 0.05 | 0.05 | 0.00 | 0.11 | 0.01 | 0.06 |
| 5 | 0.22 | 0.11 | 0.13 | 0.26 | 0.05 | 0.65 | 0.39 | 0.20 | 0.36 | 0.48 | 0.01 | 0.68 | 0.83 | 0.65 | 0.13 | 0.35 | 0.72 | 0.65 | 0.40 | 0.72 | 0.83 | 0.81 | 0.42 | 0.10 | 0.22 | 0.81 | 0.83 | 0.79 | 0.66 | 0.58 | 0.73 | 0.08 | 0.02 | 0.83 |
| 6 | 0.42 | 0.20 | 0.21 | 0.33 | 0.12 | 0.33 | 0.09 | 0.06 | 0.61 | 0.28 | 0.00 | 0.50 | 0.59 | 0.45 | 0.16 | 0.13 | 0.44 | 0.33 | 0.28 | 0.44 | 0.59 | 0.52 | 0.14 | 0.09 | 0.02 | 0.54 | 0.59 | 0.50 | 0.31 | 0.23 | 0.62 | 0.03 | 0.04 | 0.59 |
| 7 | 0.42 | 0.20 | 0.21 | 0.33 | 0.12 | 0.33 | 0.09 | 0.06 | 0.61 | 0.28 | 0.00 | 0.50 | 0.59 | 0.45 | 0.16 | 0.13 | 0.44 | 0.33 | 0.28 | 0.44 | 0.59 | 0.52 | 0.14 | 0.09 | 0.02 | 0.54 | 0.59 | 0.50 | 0.31 | 0.23 | 0.62 | 0.03 | 0.04 | 0.59 |
| 8 | 0.42 | 0.19 | 0.21 | 0.33 | 0.12 | 0.33 | 0.08 | 0.07 | 0.61 | 0.29 | 0.01 | 0.50 | 0.59 | 0.45 | 0.17 | 0.13 | 0.44 | 0.33 | 0.29 | 0.44 | 0.59 | 0.52 | 0.14 | 0.09 | 0.01 | 0.54 | 0.59 | 0.50 | 0.32 | 0.23 | 0.62 | 0.03 | 0.03 | 0.59 |
| 9 | 0.19 | 0.02 | 0.01 | 0.33 | 0.03 | 0.33 | 0.26 | 0.19 | 0.29 | 0.19 | 0.11 | 0.40 | 0.41 | 0.43 | 0.10 | 0.19 | 0.29 | 0.33 | 0.24 | 0.29 | 0.41 | 0.39 | 0.21 | 0.06 | 0.10 | 0.40 | 0.40 | 0.39 | 0.28 | 0.22 | 0.37 | 0.04 | 0.10 | 0.41 |
| 10 | 0.47 | 0.22 | 0.21 | 0.29 | 0.15 | 0.11 | 0.25 | 0.32 | 0.65 | 0.00 | 0.00 | 0.15 | 0.15 | 0.10 | 0.15 | 0.14 | 0.01 | 0.10 | 0.07 | 0.01 | 0.15 | 0.04 | 0.20 | 0.26 | 0.25 | 0.08 | 0.15 | 0.04 | 0.14 | 0.21 | 0.30 | 0.02 | 0.08 | 0.15 |
| 11 | 0.06 | 0.11 | 0.11 | 0.22 | 0.10 | 0.81 | 0.60 | 0.42 | 0.27 | 0.67 | 0.03 | 0.79 | 0.94 | 0.81 | 0.22 | 0.54 | 0.76 | 0.81 | 0.58 | 0.76 | 0.94 | 0.91 | 0.41 | 0.02 | 0.49 | 0.86 | 0.82 | 0.91 | 0.84 | 0.78 | 0.69 | 0.24 | 0.02 | 0.94 |
| 12 | 0.06 | 0.11 | 0.11 | 0.22 | 0.10 | 0.81 | 0.60 | 0.42 | 0.27 | 0.67 | 0.03 | 0.79 | 0.94 | 0.81 | 0.22 | 0.54 | 0.76 | 0.81 | 0.58 | 0.76 | 0.94 | 0.91 | 0.41 | 0.02 | 0.49 | 0.86 | 0.82 | 0.91 | 0.84 | 0.78 | 0.69 | 0.24 | 0.02 | 0.94 |
| 13 | 0.22 | 0.11 | 0.13 | 0.26 | 0.05 | 0.65 | 0.39 | 0.20 | 0.36 | 0.48 | 0.01 | 0.68 | 0.82 | 0.65 | 0.13 | 0.35 | 0.71 | 0.65 | 0.40 | 0.72 | 0.82 | 0.81 | 0.43 | 0.11 | 0.22 | 0.81 | 0.83 | 0.79 | 0.66 | 0.58 | 0.73 | 0.08 | 0.02 | 0.82 |
| 14 | 0.23 | 0.11 | 0.14 | 0.26 | 0.05 | 0.64 | 0.39 | 0.20 | 0.37 | 0.48 | 0.02 | 0.68 | 0.82 | 0.65 | 0.13 | 0.35 | 0.72 | 0.64 | 0.40 | 0.72 | 0.82 | 0.81 | 0.42 | 0.10 | 0.22 | 0.81 | 0.83 | 0.79 | 0.66 | 0.58 | 0.73 | 0.08 | 0.01 | 0.82 |
| 15 | 0.22 | 0.03 | 0.03 | 0.20 | 0.09 | 0.72 | 0.47 | 0.28 | 0.21 | 0.57 | 0.12 | 0.64 | 0.87 | 0.65 | 0.03 | 0.32 | 0.88 | 0.72 | 0.40 | 0.88 | 0.87 | 0.93 | 0.68 | 0.36 | 0.33 | 0.97 | 1.00 | 0.89 | 0.81 | 0.75 | 0.63 | 0.08 | 0.26 | 0.87 |
| 16 | 0.17 | 0.42 | 0.35 | 0.13 | 0.17 | 0.71 | 0.45 | 0.25 | 0.24 | 0.10 | 0.27 | 0.74 | 0.70 | 0.58 | 0.32 | 0.16 | 0.36 | 0.71 | 0.20 | 0.36 | 0.70 | 0.74 | 0.53 | 0.26 | 0.13 | 0.58 | 0.62 | 0.78 | 0.74 | 0.71 | 0.91 | 0.21 | 0.09 | 0.70 |
| 17 | 0.09 | 0.56 | 0.25 | 0.50 | 0.45 | 0.02 | 0.13 | 0.17 | 0.21 | 0.41 | 0.41 | 0.13 | 0.20 | 0.28 | 0.52 | 0.44 | 0.02 | 0.02 | 0.62 | 0.02 | 0.20 | 0.12 | 0.07 | 0.01 | 0.47 | 0.11 | 0.08 | 0.18 | 0.20 | 0.20 | 0.20 | 0.06 | 0.54 | 0.20 |
| 18 | 0.06 | 0.12 | 0.12 | 0.23 | 0.10 | 0.86 | 0.64 | 0.44 | 0.28 | 0.71 | 0.03 | 0.83 | 1.00 | 0.86 | 0.23 | 0.58 | 0.80 | 0.86 | 0.61 | 0.80 | 1.00 | 0.96 | 0.43 | 0.02 | 0.52 | 0.91 | 0.87 | 0.96 | 0.89 | 0.83 | 0.73 | 0.25 | 0.02 | 1.00 |
| 19 | 0.01 | 0.59 | 0.31 | 0.55 | 0.46 | 0.37 | 0.37 | 0.34 | 0.52 | 0.63 | 0.33 | 0.65 | 0.61 | 0.60 | 0.44 | 0.63 | 0.52 | 0.37 | 1.00 | 0.52 | 0.61 | 0.44 | 0.05 | 0.31 | 0.26 | 0.49 | 0.40 | 0.49 | 0.53 | 0.53 | 0.13 | 0.05 | 0.28 | 0.61 |
| 20 | 0.10 | 0.09 | 0.50 | 0.34 | 0.10 | 0.68 | 0.43 | 0.24 | 0.57 | 0.46 | 0.05 | 0.90 | 0.87 | 0.75 | 0.14 | 0.39 | 0.61 | 0.68 | 0.63 | 0.61 | 0.87 | 0.76 | 0.17 | 0.20 | 0.18 | 0.72 | 0.68 | 0.78 | 0.72 | 0.67 | 0.81 | 0.05 | 0.14 | 0.87 |
| 21 | 0.10 | 0.08 | 0.05 | 0.21 | 0.01 | 0.94 | 0.74 | 0.56 | 0.14 | 0.61 | 0.09 | 0.84 | 0.97 | 0.87 | 0.08 | 0.57 | 0.75 | 0.94 | 0.52 | 0.76 | 0.97 | 0.98 | 0.57 | 0.18 | 0.53 | 0.90 | 0.86 | 1.00 | 0.95 | 0.89 | 0.75 | 0.16 | 0.07 | 0.97 |
| 22 | 0.25 | 0.15 | 0.07 | 0.24 | 0.09 | 0.98 | 0.90 | 0.77 | 0.02 | 0.49 | 0.33 | 0.83 | 0.87 | 0.93 | 0.02 | 0.57 | 0.60 | 0.98 | 0.44 | 0.60 | 0.87 | 0.90 | 0.57 | 0.22 | 0.58 | 0.78 | 0.71 | 0.93 | 0.90 | 0.86 | 0.65 | 0.13 | 0.10 | 0.87 |
| 23 | 0.06 | 0.18 | 0.02 | 0.36 | 0.02 | 0.78 | 0.62 | 0.47 | 0.23 | 0.67 | 0.10 | 0.78 | 0.93 | 0.81 | 0.23 | 0.52 | 0.88 | 0.78 | 0.64 | 0.88 | 0.93 | 0.93 | 0.57 | 0.21 | 0.46 | 0.97 | 0.92 | 0.91 | 0.85 | 0.79 | 0.57 | 0.09 | 0.26 | 0.93 |
| 24 | 0.33 | 0.21 | 0.19 | 0.11 | 0.09 | 0.86 | 0.68 | 0.51 | 0.17 | 0.74 | 0.02 | 0.83 | 0.95 | 0.84 | 0.29 | 0.62 | 0.75 | 0.86 | 0.66 | 0.76 | 0.95 | 0.88 | 0.26 | 0.16 | 0.60 | 0.82 | 0.72 | 0.89 | 0.88 | 0.84 | 0.63 | 0.37 | 0.13 | 0.95 |
| 25 | 0.07 | 0.58 | 0.14 | 0.40 | 0.46 | 0.05 | 0.18 | 0.25 | 0.25 | 0.32 | 0.15 | 0.25 | 0.17 | 0.23 | 0.38 | 0.53 | 0.41 | 0.05 | 0.62 | 0.42 | 0.17 | 0.02 | 0.32 | 0.42 | 0.15 | 0.16 | 0.07 | 0.02 | 0.00 | 0.00 | 0.20 | 0.09 | 0.02 | 0.17 |
| 26 | 0.06 | 0.16 | 0.14 | 0.10 | 0.07 | 0.74 | 0.48 | 0.28 | 0.22 | 0.45 | 0.07 | 0.72 | 0.82 | 0.70 | 0.13 | 0.41 | 0.37 | 0.74 | 0.38 | 0.37 | 0.82 | 0.79 | 0.38 | 0.03 | 0.53 | 0.61 | 0.61 | 0.84 | 0.78 | 0.72 | 0.84 | 0.12 | 0.14 | 0.82 |
| 27 | 0.50 | 0.40 | 0.17 | 0.28 | 0.23 | 0.59 | 0.39 | 0.24 | 0.21 | 0.05 | 0.28 | 0.57 | 0.56 | 0.44 | 0.52 | 0.06 | 0.52 | 0.59 | 0.06 | 0.52 | 0.56 | 0.67 | 0.74 | 0.58 | 0.20 | 0.64 | 0.74 | 0.67 | 0.59 | 0.54 | 0.73 | 0.40 | 0.21 | 0.56 |
| 28 | 0.23 | 0.12 | 0.16 | 0.13 | 0.10 | 0.55 | 0.40 | 0.27 | 0.02 | 0.41 | 0.17 | 0.52 | 0.62 | 0.60 | 0.50 | 0.49 | 0.35 | 0.55 | 0.23 | 0.35 | 0.62 | 0.57 | 0.13 | 0.16 | 0.51 | 0.48 | 0.41 | 0.57 | 0.38 | 0.28 | 0.63 | 0.25 | 0.40 | 0.62 |
| 29 | 1.00 | 0.27 | 0.12 | 0.56 | 0.09 | 0.27 | 0.34 | 0.36 | 0.61 | 0.33 | 0.09 | 0.00 | 0.06 | 0.11 | 0.24 | 0.29 | 0.04 | 0.27 | 0.01 | 0.04 | 0.06 | 0.04 | 0.24 | 0.35 | 0.59 | 0.06 | 0.23 | 0.03 | 0.16 | 0.21 | 0.15 | 0.61 | 0.49 | 0.06 |
| 30 | 0.27 | 1.00 | 0.35 | 0.31 | 0.59 | 0.09 | 0.33 | 0.45 | 0.10 | 0.55 | 0.23 | 0.00 | 0.12 | 0.30 | 0.47 | 0.63 | 0.16 | 0.09 | 0.59 | 0.16 | 0.12 | 0.00 | 0.24 | 0.31 | 0.37 | 0.05 | 0.03 | 0.04 | 0.08 | 0.08 | 0.51 | 0.40 | 0.39 | 0.12 |
| 31 | 0.12 | 0.35 | 1.00 | 0.01 | 0.22 | 0.05 | 0.20 | 0.28 | 0.45 | 0.14 | 0.10 | 0.41 | 0.12 | 0.04 | 0.25 | 0.30 | 0.15 | 0.05 | 0.31 | 0.15 | 0.12 | 0.02 | 0.23 | 0.31 | 0.45 | 0.07 | 0.02 | 0.01 | 0.16 | 0.23 | 0.28 | 0.28 | 0.25 | 0.12 |
| 32 | 0.56 | 0.31 | 0.01 | 1.00 | 0.29 | 0.16 | 0.33 | 0.39 | 0.64 | 0.06 | 0.24 | 0.48 | 0.23 | 0.51 | 0.01 | 0.31 | 0.07 | 0.17 | 0.55 | 0.07 | 0.23 | 0.15 | 0.15 | 0.11 | 0.23 | 0.20 | 0.20 | 0.22 | 0.11 | 0.06 | 0.11 | 0.53 | 0.59 | 0.23 |
| 33 | 0.09 | 0.59 | 0.22 | 0.29 | 1.00 | 0.11 | 0.09 | 0.05 | 0.34 | 0.42 | 0.36 | 0.08 | 0.10 | 0.08 | 0.65 | 0.69 | 0.05 | 0.11 | 0.46 | 0.05 | 0.10 | 0.07 | 0.45 | 0.52 | 0.12 | 0.14 | 0.08 | 0.00 | 0.14 | 0.19 | 0.12 | 0.30 | 0.04 | 0.10 |
| 34 | 0.27 | 0.09 | 0.05 | 0.16 | 0.11 | 1.00 | 0.90 | 0.76 | 0.04 | 0.46 | 0.37 | 0.81 | 0.86 | 0.89 | 0.08 | 0.56 | 0.64 | 1.00 | 0.37 | 0.65 | 0.86 | 0.90 | 0.57 | 0.22 | 0.51 | 0.79 | 0.72 | 0.92 | 0.89 | 0.84 | 0.69 | 0.15 | 0.01 | 0.86 |
| 35 | 0.34 | 0.33 | 0.20 | 0.33 | 0.09 | 0.90 | 1.00 | 0.97 | 0.12 | 0.30 | 0.54 | 0.70 | 0.64 | 0.87 | 0.14 | 0.60 | 0.45 | 0.90 | 0.37 | 0.45 | 0.64 | 0.67 | 0.49 | 0.24 | 0.44 | 0.57 | 0.48 | 0.72 | 0.71 | 0.68 | 0.39 | 0.04 | 0.18 | 0.64 |
| 36 | 0.36 | 0.45 | 0.28 | 0.39 | 0.05 | 0.76 | 0.97 | 1.00 | 0.15 | 0.19 | 0.58 | 0.57 | 0.44 | 0.77 | 0.16 | 0.57 | 0.29 | 0.76 | 0.34 | 0.29 | 0.44 | 0.47 | 0.39 | 0.22 | 0.36 | 0.39 | 0.28 | 0.53 | 0.54 | 0.52 | 0.18 | 0.01 | 0.27 | 0.44 |
| 37 | 0.61 | 0.10 | 0.45 | 0.64 | 0.34 | 0.04 | 0.12 | 0.15 | 1.00 | 0.10 | 0.07 | 0.41 | 0.28 | 0.30 | 0.12 | 0.03 | 0.10 | 0.03 | 0.52 | 0.10 | 0.28 | 0.10 | 0.25 | 0.35 | 0.33 | 0.17 | 0.21 | 0.14 | 0.03 | 0.01 | 0.24 | 0.16 | 0.23 | 0.28 |
| 38 | 0.33 | 0.55 | 0.14 | 0.06 | 0.42 | 0.46 | 0.30 | 0.19 | 0.10 | 1.00 | 0.53 | 0.32 | 0.71 | 0.49 | 0.70 | 0.64 | 0.69 | 0.46 | 0.63 | 0.69 | 0.71 | 0.61 | 0.02 | 0.30 | 0.74 | 0.63 | 0.57 | 0.59 | 0.57 | 0.54 | 0.14 | 0.71 | 0.01 | 0.71 |
| 39 | 0.09 | 0.23 | 0.10 | 0.24 | 0.36 | 0.37 | 0.54 | 0.58 | 0.07 | 0.53 | 1.00 | 0.29 | 0.03 | 0.35 | 0.57 | 0.04 | 0.25 | 0.37 | 0.33 | 0.25 | 0.03 | 0.03 | 0.18 | 0.20 | 0.21 | 0.10 | 0.12 | 0.08 | 0.00 | 0.03 | 0.31 | 0.29 | 0.11 | 0.03 |
| 40 | 0.00 | 0.00 | 0.41 | 0.48 | 0.08 | 0.81 | 0.70 | 0.57 | 0.41 | 0.32 | 0.29 | 1.00 | 0.83 | 0.87 | 0.06 | 0.43 | 0.59 | 0.81 | 0.65 | 0.59 | 0.83 | 0.77 | 0.35 | 0.01 | 0.19 | 0.73 | 0.64 | 0.81 | 0.80 | 0.77 | 0.73 | 0.15 | 0.04 | 0.83 |
| 41 | 0.06 | 0.12 | 0.12 | 0.23 | 0.10 | 0.86 | 0.64 | 0.44 | 0.28 | 0.71 | 0.03 | 0.83 | 1.00 | 0.86 | 0.23 | 0.58 | 0.80 | 0.86 | 0.61 | 0.80 | 1.00 | 0.96 | 0.43 | 0.02 | 0.52 | 0.91 | 0.87 | 0.96 | 0.89 | 0.83 | 0.73 | 0.25 | 0.02 | 1.00 |
| 42 | 0.11 | 0.30 | 0.04 | 0.51 | 0.08 | 0.89 | 0.87 | 0.77 | 0.30 | 0.49 | 0.35 | 0.87 | 0.86 | 1.00 | 0.11 | 0.63 | 0.56 | 0.89 | 0.60 | 0.56 | 0.86 | 0.81 | 0.38 | 0.03 | 0.46 | 0.73 | 0.66 | 0.85 | 0.77 | 0.70 | 0.59 | 0.10 | 0.17 | 0.86 |
| 43 | 0.24 | 0.47 | 0.25 | 0.01 | 0.65 | 0.08 | 0.14 | 0.16 | 0.12 | 0.70 | 0.57 | 0.06 | 0.23 | 0.11 | 1.00 | 0.54 | 0.16 | 0.08 | 0.44 | 0.16 | 0.23 | 0.07 | 0.44 | 0.59 | 0.54 | 0.08 | 0.03 | 0.06 | 0.05 | 0.10 | 0.13 | 0.60 | 0.21 | 0.23 |
| 44 | 0.29 | 0.63 | 0.30 | 0.31 | 0.69 | 0.56 | 0.60 | 0.57 | 0.03 | 0.64 | 0.04 | 0.43 | 0.58 | 0.63 | 0.54 | 1.00 | 0.39 | 0.56 | 0.63 | 0.39 | 0.58 | 0.48 | 0.02 | 0.24 | 0.51 | 0.37 | 0.32 | 0.55 | 0.45 | 0.39 | 0.21 | 0.33 | 0.03 | 0.58 |
| 45 | 0.04 | 0.16 | 0.15 | 0.07 | 0.05 | 0.64 | 0.45 | 0.29 | 0.10 | 0.69 | 0.25 | 0.59 | 0.80 | 0.56 | 0.16 | 0.39 | 1.00 | 0.64 | 0.52 | 1.00 | 0.80 | 0.81 | 0.44 | 0.12 | 0.31 | 0.92 | 0.88 | 0.75 | 0.75 | 0.72 | 0.41 | 0.25 | 0.11 | 0.80 |
| 46 | 0.27 | 0.09 | 0.05 | 0.17 | 0.11 | 1.00 | 0.90 | 0.76 | 0.03 | 0.46 | 0.37 | 0.81 | 0.86 | 0.89 | 0.08 | 0.56 | 0.64 | 1.00 | 0.37 | 0.65 | 0.86 | 0.90 | 0.57 | 0.22 | 0.51 | 0.79 | 0.72 | 0.92 | 0.89 | 0.84 | 0.69 | 0.15 | 0.01 | 0.86 |
| 47 | 0.01 | 0.59 | 0.31 | 0.55 | 0.46 | 0.37 | 0.37 | 0.34 | 0.52 | 0.63 | 0.33 | 0.65 | 0.61 | 0.60 | 0.44 | 0.63 | 0.52 | 0.37 | 1.00 | 0.52 | 0.61 | 0.44 | 0.05 | 0.31 | 0.26 | 0.49 | 0.40 | 0.49 | 0.53 | 0.53 | 0.13 | 0.05 | 0.28 | 0.61 |
| 48 | 0.04 | 0.16 | 0.15 | 0.07 | 0.05 | 0.65 | 0.45 | 0.29 | 0.10 | 0.69 | 0.25 | 0.59 | 0.80 | 0.56 | 0.16 | 0.39 | 1.00 | 0.65 | 0.52 | 1.00 | 0.80 | 0.82 | 0.44 | 0.12 | 0.31 | 0.92 | 0.88 | 0.75 | 0.75 | 0.72 | 0.42 | 0.25 | 0.11 | 0.80 |
| 49 | 0.06 | 0.12 | 0.12 | 0.23 | 0.10 | 0.86 | 0.64 | 0.44 | 0.28 | 0.71 | 0.03 | 0.83 | 1.00 | 0.86 | 0.23 | 0.58 | 0.80 | 0.86 | 0.61 | 0.80 | 1.00 | 0.96 | 0.43 | 0.02 | 0.52 | 0.91 | 0.87 | 0.96 | 0.89 | 0.83 | 0.73 | 0.25 | 0.02 | 1.00 |
| 50 | 0.04 | 0.00 | 0.02 | 0.15 | 0.07 | 0.90 | 0.67 | 0.47 | 0.10 | 0.61 | 0.03 | 0.77 | 0.96 | 0.81 | 0.07 | 0.48 | 0.81 | 0.90 | 0.44 | 0.82 | 0.96 | 1.00 | 0.65 | 0.27 | 0.52 | 0.95 | 0.93 | 0.99 | 0.93 | 0.87 | 0.74 | 0.16 | 0.09 | 0.96 |
| 51 | 0.24 | 0.24 | 0.23 | 0.15 | 0.45 | 0.57 | 0.49 | 0.39 | 0.25 | 0.02 | 0.18 | 0.35 | 0.43 | 0.38 | 0.44 | 0.02 | 0.44 | 0.57 | 0.05 | 0.44 | 0.43 | 0.65 | 1.00 | 0.91 | 0.20 | 0.63 | 0.68 | 0.63 | 0.63 | 0.61 | 0.43 | 0.37 | 0.48 | 0.43 |
| 52 | 0.35 | 0.31 | 0.31 | 0.11 | 0.52 | 0.22 | 0.24 | 0.22 | 0.35 | 0.30 | 0.20 | 0.01 | 0.02 | 0.03 | 0.59 | 0.24 | 0.12 | 0.22 | 0.31 | 0.12 | 0.02 | 0.27 | 0.91 | 1.00 | 0.04 | 0.29 | 0.37 | 0.25 | 0.28 | 0.28 | 0.13 | 0.55 | 0.57 | 0.02 |
| 53 | 0.59 | 0.37 | 0.45 | 0.23 | 0.12 | 0.51 | 0.44 | 0.36 | 0.33 | 0.74 | 0.21 | 0.19 | 0.52 | 0.46 | 0.54 | 0.51 | 0.31 | 0.51 | 0.26 | 0.31 | 0.52 | 0.52 | 0.20 | 0.04 | 1.00 | 0.42 | 0.33 | 0.52 | 0.52 | 0.50 | 0.13 | 0.63 | 0.03 | 0.52 |
| 54 | 0.06 | 0.05 | 0.07 | 0.20 | 0.14 | 0.79 | 0.57 | 0.39 | 0.17 | 0.63 | 0.10 | 0.73 | 0.91 | 0.73 | 0.08 | 0.37 | 0.92 | 0.79 | 0.49 | 0.92 | 0.91 | 0.95 | 0.63 | 0.29 | 0.42 | 1.00 | 0.97 | 0.91 | 0.87 | 0.82 | 0.60 | 0.13 | 0.22 | 0.91 |
| 55 | 0.23 | 0.03 | 0.02 | 0.20 | 0.08 | 0.72 | 0.48 | 0.28 | 0.21 | 0.57 | 0.12 | 0.64 | 0.87 | 0.66 | 0.03 | 0.32 | 0.88 | 0.72 | 0.40 | 0.88 | 0.87 | 0.93 | 0.68 | 0.37 | 0.33 | 0.97 | 1.00 | 0.89 | 0.81 | 0.75 | 0.64 | 0.08 | 0.26 | 0.87 |
| 56 | 0.03 | 0.04 | 0.01 | 0.22 | 0.00 | 0.92 | 0.72 | 0.53 | 0.14 | 0.59 | 0.08 | 0.81 | 0.96 | 0.85 | 0.06 | 0.55 | 0.75 | 0.92 | 0.49 | 0.75 | 0.96 | 0.99 | 0.63 | 0.25 | 0.52 | 0.91 | 0.89 | 1.00 | 0.94 | 0.88 | 0.76 | 0.13 | 0.11 | 0.96 |
| 57 | 0.16 | 0.08 | 0.16 | 0.11 | 0.14 | 0.89 | 0.71 | 0.54 | 0.03 | 0.57 | 0.00 | 0.80 | 0.89 | 0.77 | 0.05 | 0.45 | 0.75 | 0.89 | 0.53 | 0.75 | 0.89 | 0.93 | 0.63 | 0.28 | 0.52 | 0.87 | 0.81 | 0.94 | 1.00 | 0.99 | 0.63 | 0.08 | 0.16 | 0.89 |
| 58 | 0.21 | 0.08 | 0.23 | 0.06 | 0.19 | 0.84 | 0.68 | 0.52 | 0.01 | 0.54 | 0.03 | 0.77 | 0.83 | 0.70 | 0.10 | 0.39 | 0.72 | 0.84 | 0.53 | 0.72 | 0.83 | 0.87 | 0.61 | 0.28 | 0.50 | 0.82 | 0.75 | 0.88 | 0.99 | 1.00 | 0.55 | 0.05 | 0.17 | 0.83 |
| 59 | 0.15 | 0.51 | 0.28 | 0.11 | 0.12 | 0.69 | 0.39 | 0.18 | 0.24 | 0.14 | 0.31 | 0.73 | 0.73 | 0.59 | 0.13 | 0.21 | 0.41 | 0.69 | 0.13 | 0.42 | 0.73 | 0.74 | 0.43 | 0.13 | 0.13 | 0.60 | 0.64 | 0.76 | 0.63 | 0.55 | 1.00 | 0.10 | 0.31 | 0.73 |
| 60 | 0.61 | 0.40 | 0.28 | 0.53 | 0.30 | 0.15 | 0.04 | 0.01 | 0.16 | 0.71 | 0.29 | 0.15 | 0.25 | 0.10 | 0.60 | 0.33 | 0.25 | 0.15 | 0.05 | 0.25 | 0.25 | 0.16 | 0.37 | 0.55 | 0.63 | 0.13 | 0.08 | 0.13 | 0.08 | 0.05 | 0.10 | 1.00 | 0.40 | 0.25 |
| 61 | 0.49 | 0.39 | 0.25 | 0.59 | 0.04 | 0.01 | 0.18 | 0.27 | 0.23 | 0.01 | 0.11 | 0.04 | 0.02 | 0.17 | 0.21 | 0.03 | 0.11 | 0.01 | 0.28 | 0.11 | 0.02 | 0.09 | 0.48 | 0.57 | 0.03 | 0.22 | 0.26 | 0.11 | 0.16 | 0.17 | 0.31 | 0.40 | 1.00 | 0.02 |
| 62 | 0.06 | 0.12 | 0.12 | 0.23 | 0.10 | 0.86 | 0.64 | 0.44 | 0.28 | 0.71 | 0.03 | 0.83 | 1.00 | 0.86 | 0.23 | 0.58 | 0.80 | 0.86 | 0.61 | 0.80 | 1.00 | 0.96 | 0.43 | 0.02 | 0.52 | 0.91 | 0.87 | 0.96 | 0.89 | 0.83 | 0.73 | 0.25 | 0.02 | 1.00 |

**Key:**

| |
|---|
| 1.00 |
| 0.95 |
| 0.90 |
| 0.85 |
| 0.80 |
| 0.75 |
| 0.70 |
| 0.65 |
| 0.60 |
| 0.55 |
| 0.50 |
| 0.45 |
| 0.40 |
| 0.35 |
| 0.30 |
| 0.25 |
| 0.20 |
| 0.15 |
| 0.10 |
| 0.05 |
| 0.00 |

## All properties with correlation coefficient < 0.9

| | 4 | 8 | 9 | 10 | 14 | 15 | 16 | 17 | 19 | 21 | 25 | 26 | 27 | 28 | 29 | 30 | 31 | 32 | 33 | 36 | 37 | 38 | 39 | 40 | 42 | 43 | 44 | 48 | 52 | 53 | 58 | 60 | 61 |
|---|---|---|---|---|---|---|---|---|---|---|---|---|---|---|---|---|---|---|---|---|---|---|---|---|---|---|---|---|---|---|---|---|---|
| 4 | 1.00 | 0.06 | 0.22 | 0.13 | 0.03 | 0.06 | 0.01 | 0.06 | 0.01 | 0.05 | 0.03 | 0.05 | 0.03 | 0.06 | 0.07 | 0.04 | 0.09 | 0.08 | 0.03 | 0.01 | 0.08 | 0.13 | 0.10 | 0.03 | 0.01 | 0.10 | 0.06 | 0.06 | 0.01 | 0.15 | 0.05 | 0.11 | 0.01 |
| 8 | 0.06 | 1.00 | 0.53 | 0.83 | 0.84 | 0.60 | 0.48 | 0.02 | 0.29 | 0.49 | 0.08 | 0.48 | 0.49 | 0.46 | 0.42 | 0.19 | 0.21 | 0.33 | 0.12 | 0.07 | 0.61 | 0.29 | 0.01 | 0.50 | 0.45 | 0.17 | 0.13 | 0.44 | 0.09 | 0.01 | 0.23 | 0.03 | 0.03 |
| 9 | 0.22 | 0.53 | 1.00 | 0.37 | 0.52 | 0.40 | 0.29 | 0.11 | 0.24 | 0.39 | 0.09 | 0.33 | 0.30 | 0.37 | 0.19 | 0.02 | 0.01 | 0.33 | 0.03 | 0.19 | 0.29 | 0.19 | 0.11 | 0.40 | 0.43 | 0.10 | 0.19 | 0.29 | 0.06 | 0.10 | 0.22 | 0.04 | 0.10 |
| 10 | 0.13 | 0.83 | 0.37 | 1.00 | 0.38 | 0.15 | 0.15 | 0.06 | 0.07 | 0.03 | 0.01 | 0.15 | 0.15 | 0.22 | 0.47 | 0.22 | 0.21 | 0.29 | 0.15 | 0.32 | 0.65 | 0.00 | 0.00 | 0.15 | 0.10 | 0.15 | 0.14 | 0.01 | 0.26 | 0.25 | 0.21 | 0.02 | 0.08 |
| 14 | 0.03 | 0.84 | 0.52 | 0.38 | 1.00 | 0.83 | 0.63 | 0.02 | 0.40 | 0.78 | 0.13 | 0.64 | 0.65 | 0.54 | 0.23 | 0.11 | 0.14 | 0.26 | 0.05 | 0.20 | 0.37 | 0.48 | 0.02 | 0.68 | 0.65 | 0.13 | 0.35 | 0.72 | 0.10 | 0.22 | 0.58 | 0.08 | 0.01 |
| 15 | 0.06 | 0.60 | 0.40 | 0.15 | 0.83 | 1.00 | 0.62 | 0.08 | 0.40 | 0.86 | 0.07 | 0.60 | 0.74 | 0.41 | 0.22 | 0.03 | 0.03 | 0.20 | 0.09 | 0.28 | 0.21 | 0.57 | 0.12 | 0.64 | 0.65 | 0.03 | 0.32 | 0.88 | 0.36 | 0.33 | 0.75 | 0.08 | 0.26 |
| 16 | 0.01 | 0.48 | 0.29 | 0.15 | 0.63 | 0.62 | 1.00 | 0.08 | 0.20 | 0.77 | 0.33 | 0.87 | 0.78 | 0.36 | 0.17 | 0.42 | 0.35 | 0.13 | 0.17 | 0.25 | 0.24 | 0.10 | 0.27 | 0.74 | 0.58 | 0.32 | 0.16 | 0.36 | 0.26 | 0.13 | 0.71 | 0.21 | 0.09 |
| 17 | 0.06 | 0.02 | 0.11 | 0.06 | 0.02 | 0.08 | 0.08 | 1.00 | 0.62 | 0.18 | 0.11 | 0.24 | 0.26 | 0.12 | 0.09 | 0.56 | 0.25 | 0.50 | 0.45 | 0.17 | 0.21 | 0.41 | 0.41 | 0.13 | 0.28 | 0.52 | 0.44 | 0.02 | 0.01 | 0.47 | 0.20 | 0.06 | 0.54 |
| 19 | 0.01 | 0.29 | 0.24 | 0.07 | 0.40 | 0.40 | 0.20 | 0.62 | 1.00 | 0.52 | 0.62 | 0.38 | 0.06 | 0.23 | 0.01 | 0.59 | 0.31 | 0.55 | 0.46 | 0.34 | 0.52 | 0.63 | 0.33 | 0.65 | 0.60 | 0.44 | 0.63 | 0.52 | 0.31 | 0.26 | 0.53 | 0.05 | 0.28 |
| 21 | 0.05 | 0.49 | 0.39 | 0.03 | 0.78 | 0.86 | 0.77 | 0.18 | 0.52 | 1.00 | 0.06 | 0.84 | 0.63 | 0.58 | 0.10 | 0.08 | 0.05 | 0.21 | 0.01 | 0.56 | 0.14 | 0.61 | 0.09 | 0.84 | 0.87 | 0.08 | 0.57 | 0.76 | 0.18 | 0.53 | 0.89 | 0.16 | 0.07 |
| 25 | 0.03 | 0.08 | 0.09 | 0.01 | 0.13 | 0.07 | 0.33 | 0.11 | 0.62 | 0.06 | 1.00 | 0.28 | 0.12 | 0.11 | 0.07 | 0.58 | 0.14 | 0.40 | 0.46 | 0.25 | 0.25 | 0.32 | 0.15 | 0.25 | 0.23 | 0.38 | 0.53 | 0.42 | 0.42 | 0.15 | 0.00 | 0.09 | 0.02 |
| 26 | 0.05 | 0.48 | 0.33 | 0.15 | 0.64 | 0.60 | 0.87 | 0.24 | 0.38 | 0.84 | 0.28 | 1.00 | 0.47 | 0.61 | 0.06 | 0.16 | 0.14 | 0.10 | 0.07 | 0.28 | 0.22 | 0.45 | 0.07 | 0.72 | 0.70 | 0.13 | 0.41 | 0.37 | 0.03 | 0.53 | 0.72 | 0.12 | 0.14 |
| 27 | 0.03 | 0.49 | 0.30 | 0.15 | 0.65 | 0.74 | 0.78 | 0.26 | 0.06 | 0.63 | 0.12 | 0.47 | 1.00 | 0.13 | 0.50 | 0.40 | 0.17 | 0.28 | 0.23 | 0.24 | 0.21 | 0.05 | 0.28 | 0.57 | 0.44 | 0.52 | 0.06 | 0.52 | 0.58 | 0.20 | 0.54 | 0.40 | 0.21 |
| 28 | 0.06 | 0.46 | 0.37 | 0.22 | 0.54 | 0.41 | 0.36 | 0.12 | 0.23 | 0.58 | 0.11 | 0.61 | 0.13 | 1.00 | 0.23 | 0.12 | 0.16 | 0.13 | 0.10 | 0.27 | 0.02 | 0.41 | 0.17 | 0.52 | 0.60 | 0.50 | 0.49 | 0.35 | 0.16 | 0.51 | 0.28 | 0.25 | 0.40 |
| 29 | 0.07 | 0.42 | 0.19 | 0.47 | 0.23 | 0.22 | 0.17 | 0.09 | 0.01 | 0.10 | 0.07 | 0.06 | 0.50 | 0.23 | 1.00 | 0.27 | 0.12 | 0.56 | 0.09 | 0.36 | 0.61 | 0.33 | 0.09 | 0.00 | 0.11 | 0.24 | 0.29 | 0.04 | 0.35 | 0.59 | 0.21 | 0.61 | 0.49 |
| 30 | 0.04 | 0.19 | 0.02 | 0.22 | 0.11 | 0.03 | 0.42 | 0.56 | 0.59 | 0.08 | 0.58 | 0.16 | 0.40 | 0.12 | 0.27 | 1.00 | 0.35 | 0.31 | 0.59 | 0.45 | 0.10 | 0.55 | 0.23 | 0.00 | 0.30 | 0.47 | 0.63 | 0.16 | 0.31 | 0.37 | 0.08 | 0.40 | 0.39 |
| 31 | 0.09 | 0.21 | 0.01 | 0.21 | 0.14 | 0.03 | 0.35 | 0.25 | 0.31 | 0.05 | 0.14 | 0.14 | 0.17 | 0.16 | 0.12 | 0.35 | 1.00 | 0.01 | 0.22 | 0.28 | 0.45 | 0.14 | 0.10 | 0.41 | 0.04 | 0.25 | 0.30 | 0.15 | 0.31 | 0.45 | 0.23 | 0.28 | 0.25 |
| 32 | 0.08 | 0.33 | 0.33 | 0.29 | 0.26 | 0.20 | 0.13 | 0.50 | 0.55 | 0.21 | 0.40 | 0.10 | 0.28 | 0.13 | 0.56 | 0.31 | 0.01 | 1.00 | 0.29 | 0.39 | 0.64 | 0.06 | 0.24 | 0.48 | 0.51 | 0.01 | 0.31 | 0.07 | 0.11 | 0.23 | 0.06 | 0.53 | 0.59 |
| 33 | 0.03 | 0.12 | 0.03 | 0.15 | 0.05 | 0.09 | 0.17 | 0.45 | 0.46 | 0.01 | 0.46 | 0.07 | 0.23 | 0.10 | 0.09 | 0.59 | 0.22 | 0.29 | 1.00 | 0.05 | 0.34 | 0.42 | 0.36 | 0.08 | 0.08 | 0.65 | 0.69 | 0.05 | 0.52 | 0.12 | 0.19 | 0.30 | 0.04 |
| 36 | 0.01 | 0.07 | 0.19 | 0.32 | 0.20 | 0.28 | 0.25 | 0.17 | 0.34 | 0.56 | 0.25 | 0.28 | 0.24 | 0.27 | 0.36 | 0.45 | 0.28 | 0.39 | 0.05 | 1.00 | 0.15 | 0.19 | 0.58 | 0.57 | 0.77 | 0.16 | 0.57 | 0.29 | 0.22 | 0.36 | 0.52 | 0.01 | 0.27 |
| 37 | 0.08 | 0.61 | 0.29 | 0.65 | 0.37 | 0.21 | 0.24 | 0.21 | 0.52 | 0.14 | 0.25 | 0.22 | 0.21 | 0.02 | 0.61 | 0.10 | 0.45 | 0.64 | 0.34 | 0.15 | 1.00 | 0.10 | 0.07 | 0.41 | 0.30 | 0.12 | 0.03 | 0.10 | 0.35 | 0.33 | 0.01 | 0.16 | 0.23 |
| 38 | 0.13 | 0.29 | 0.19 | 0.00 | 0.48 | 0.57 | 0.10 | 0.41 | 0.63 | 0.61 | 0.32 | 0.45 | 0.05 | 0.41 | 0.33 | 0.55 | 0.14 | 0.06 | 0.42 | 0.19 | 0.10 | 1.00 | 0.53 | 0.32 | 0.49 | 0.70 | 0.64 | 0.69 | 0.30 | 0.74 | 0.54 | 0.71 | 0.01 |
| 39 | 0.10 | 0.01 | 0.11 | 0.00 | 0.02 | 0.12 | 0.27 | 0.41 | 0.33 | 0.09 | 0.15 | 0.07 | 0.28 | 0.17 | 0.09 | 0.23 | 0.10 | 0.24 | 0.36 | 0.58 | 0.07 | 0.53 | 1.00 | 0.29 | 0.35 | 0.57 | 0.04 | 0.25 | 0.20 | 0.21 | 0.03 | 0.29 | 0.11 |
| 40 | 0.03 | 0.50 | 0.40 | 0.15 | 0.68 | 0.64 | 0.74 | 0.13 | 0.65 | 0.84 | 0.25 | 0.72 | 0.57 | 0.52 | 0.00 | 0.00 | 0.41 | 0.48 | 0.08 | 0.57 | 0.41 | 0.32 | 0.29 | 1.00 | 0.87 | 0.06 | 0.43 | 0.59 | 0.01 | 0.19 | 0.77 | 0.15 | 0.04 |
| 42 | 0.01 | 0.45 | 0.43 | 0.10 | 0.65 | 0.65 | 0.58 | 0.28 | 0.60 | 0.87 | 0.23 | 0.70 | 0.44 | 0.60 | 0.11 | 0.30 | 0.04 | 0.51 | 0.08 | 0.77 | 0.30 | 0.49 | 0.35 | 0.87 | 1.00 | 0.11 | 0.63 | 0.56 | 0.03 | 0.46 | 0.70 | 0.10 | 0.17 |
| 43 | 0.10 | 0.17 | 0.10 | 0.15 | 0.13 | 0.03 | 0.32 | 0.52 | 0.44 | 0.08 | 0.38 | 0.13 | 0.52 | 0.50 | 0.24 | 0.47 | 0.25 | 0.01 | 0.65 | 0.16 | 0.12 | 0.70 | 0.57 | 0.06 | 0.11 | 1.00 | 0.54 | 0.16 | 0.59 | 0.54 | 0.10 | 0.60 | 0.21 |
| 44 | 0.06 | 0.13 | 0.19 | 0.14 | 0.35 | 0.32 | 0.16 | 0.44 | 0.63 | 0.57 | 0.53 | 0.41 | 0.06 | 0.49 | 0.29 | 0.63 | 0.30 | 0.31 | 0.69 | 0.57 | 0.03 | 0.64 | 0.04 | 0.43 | 0.63 | 0.54 | 1.00 | 0.39 | 0.24 | 0.51 | 0.39 | 0.33 | 0.03 |
| 48 | 0.06 | 0.44 | 0.29 | 0.01 | 0.72 | 0.88 | 0.36 | 0.02 | 0.52 | 0.76 | 0.42 | 0.37 | 0.52 | 0.35 | 0.04 | 0.16 | 0.15 | 0.07 | 0.05 | 0.29 | 0.10 | 0.69 | 0.25 | 0.59 | 0.56 | 0.16 | 0.39 | 1.00 | 0.12 | 0.31 | 0.72 | 0.25 | 0.11 |
| 52 | 0.01 | 0.09 | 0.06 | 0.26 | 0.10 | 0.36 | 0.26 | 0.01 | 0.31 | 0.18 | 0.42 | 0.03 | 0.58 | 0.16 | 0.35 | 0.31 | 0.31 | 0.11 | 0.52 | 0.22 | 0.35 | 0.30 | 0.20 | 0.01 | 0.03 | 0.59 | 0.24 | 0.12 | 1.00 | 0.04 | 0.28 | 0.55 | 0.57 |
| 53 | 0.15 | 0.01 | 0.10 | 0.25 | 0.22 | 0.33 | 0.13 | 0.47 | 0.26 | 0.53 | 0.15 | 0.53 | 0.20 | 0.51 | 0.59 | 0.37 | 0.45 | 0.23 | 0.12 | 0.36 | 0.33 | 0.74 | 0.21 | 0.19 | 0.46 | 0.54 | 0.51 | 0.31 | 0.04 | 1.00 | 0.50 | 0.63 | 0.03 |
| 58 | 0.05 | 0.23 | 0.22 | 0.21 | 0.58 | 0.75 | 0.71 | 0.20 | 0.53 | 0.89 | 0.00 | 0.72 | 0.54 | 0.28 | 0.21 | 0.08 | 0.23 | 0.06 | 0.19 | 0.52 | 0.01 | 0.54 | 0.03 | 0.77 | 0.70 | 0.10 | 0.39 | 0.72 | 0.28 | 0.50 | 1.00 | 0.05 | 0.17 |
| 60 | 0.11 | 0.03 | 0.04 | 0.02 | 0.08 | 0.08 | 0.21 | 0.06 | 0.05 | 0.16 | 0.09 | 0.12 | 0.40 | 0.25 | 0.61 | 0.40 | 0.28 | 0.53 | 0.30 | 0.01 | 0.16 | 0.71 | 0.29 | 0.15 | 0.10 | 0.60 | 0.33 | 0.25 | 0.55 | 0.63 | 0.05 | 1.00 | 0.40 |
| 61 | 0.01 | 0.03 | 0.10 | 0.08 | 0.01 | 0.26 | 0.09 | 0.54 | 0.28 | 0.07 | 0.02 | 0.14 | 0.21 | 0.40 | 0.49 | 0.39 | 0.25 | 0.59 | 0.04 | 0.27 | 0.23 | 0.01 | 0.11 | 0.04 | 0.17 | 0.21 | 0.03 | 0.11 | 0.57 | 0.03 | 0.17 | 0.40 | 1.00 |
